# Development of Value-Added Chicken Burgers by Adding Pumpkin Peel Powder as a Sustainable Ingredient

**DOI:** 10.3390/antiox14060648

**Published:** 2025-05-28

**Authors:** Nicola Pinna, Federica Ianni, Michela Codini, Beniamino Terzo Cenci-Goga, Marco Misuraca, Egidia Costanzi, Lina Cossignani, Francesca Blasi

**Affiliations:** 1 Department of Pharmaceutical Sciences, University of Perugia, 06126 Perugia, Italy; nicola.pinna@dottorandi.unipg.it (N.P.); federica.ianni@unipg.it (F.I.); michela.codini@unipg.it (M.C.); francesca.blasi@unipg.it (F.B.); 2 Department of Veterinary Medicine, University of Perugia, 06126 Perugia, Italy; beniamino.cencigoga@unipg.it (B.T.C.-G.); marco.misuraca.tdp@gmail.com (M.M.); egidia.costanzi@unipg.it (E.C.); 3 Department of Paraclinical Sciences, Faculty of Veterinary Science, University of Pretoria, Onderstepoort 0110, South Africa

**Keywords:** agri-food waste, carotenoids, ultrasound-assisted extraction, fortified meat, microbiological assay, sensory analysis

## Abstract

Worldwide, there is a growing need to valorize agri-food waste containing bioactive compounds to fit into the circular economy action plan approved in Europe. In this paper, the carotenoids of peel powder of pumpkins (PPP) of five varieties (Hokkaido, Lunga di Napoli, Mantovana, Moscata di Provenza, and Violina rugosa) were characterized by spectrophotometric (antioxidant activity) and chromatographic analyses. PPP from the Hokkaido variety showed high levels of carotenoids (2993.90 μg β-carotene equivalents/g). They were mainly composed of mono- (9065.35 μg zeaxanthin dipalmitate equivalents/g) and di-esterified (1832.74 μg zeaxanthin dipalmitate equivalents/g) xanthophylls. It also showed high antioxidant activity (ABTS 2036.02 μg Trolox equivalents/g). Therefore, it was used as a functional plant ingredient (4%) to prepare chicken burgers (100, 70, and 50% chicken meat). Physical-chemical, microbiological, color, and sensorial analyses of fortified chicken burgers were carried out. The product with 70% chicken meat and 4% PPP obtained the highest overall acceptability score (5.95 ± 0.25). The results confirm that the addition of PPP could represent a valid approach to increasing the health properties and acceptability of burgers, even if a larger assessor size is necessary.

## 1. Introduction

Over 59 million tons of agri-food waste are generated in the European Union (EU) every year. Households generate more than half of the total (54%), while the remaining 46% is generated upstream in the food supply chain, resulting in significant environmental damage and financial costs [1].

It should also be noted that by-products, such as peels, stems, shells, and seeds, often have higher nutritional and functional properties than the edible parts [2]. Turning waste and by-products into value-added products is an essential challenge for economic, social, and environmental reasons. From the perspective of a circular economy, they can be valuable resources for recovering functional bioactive compounds (i.e., phenolic compounds, carotenoids) to be used as natural additives/ingredients for the production of value-added foods, supplements, or nutraceuticals [3]. Recently, it has been reported that the functional constituents of vegetable foods can enhance immunity against various disorders (acute and chronic) [4]. Pumpkin is used in numerous fields as a functional food, including beverages, baking, and dairy products [5]. In terms of phytochemical composition, pumpkin pulp, as well as seeds and peels, often discarded as by-products, contain numerous bioactive compounds, including carotenoids, vitamin E, ascorbic acid, phytosterols, and selenium [6]. Interestingly, some researchers reported the potential food utilization of different forms of pumpkin by-products (peel and seed flour) into plant-based and meat foodstuffs [7]. As people of all ages from different cultures enjoy various ground meals, including meatballs, sausages, and burgers, the replacement of meat-based products with plant-based foods can provide many valuable benefits. These alternatives can be crafted to resemble meat in texture, taste, and appearance, while potentially being lower in fat and calories, and higher in fiber. Generally, the reduction of fat in meat products has several positive repercussions on nutritional, sensory, and technical aspects [8], as well as an antioxidant-rich diet reduces the incidence of diabetes, cancer, and cardiovascular disease [9]. A reformulation of meat products represents an interesting challenge for the researchers, incorporating natural antioxidants and emulsifiers, adding fiber sources, and reducing salt levels and sodium nitrite content [10,11,12]. For example, the addition of pumpkin seed kernel flour improved the quality of beef burgers [13] and beef meatballs [14]. Haddad et al. suggested the use of fresh pumpkin fruits for producing chicken sausage with functional properties [15], while Longato et al. used pumpkin seeds to improve the quality of chicken burgers [11]. The chemical composition of horse meat cutlets with added pumpkin was also studied [16].

This research also aims to develop a functional food made with agri-food waste and endowed with economic feasibility. Different from other papers [11,12,13,14,15,16], initially, the carotenoids of pumpkin peel powder (PPP) of five varieties were deeply characterized by spectrophotometric assays and chromatographic analysis. Among them, PPP from Hokkaido (4%) was chosen to prepare chicken burgers (100, 70, and 50% of chicken meat). Their physical-chemical properties, color, microbiological characteristics, and sensory and textural properties were evaluated. The inclusion of PPP in these meat products may ameliorate their quality, aligning with the European zero-waste approach and healthy diet.

## 2. Materials and Methods

### 2.1. Plant Materials and Reagents

Fresh whole pumpkins (*Cucurbita* spp.) of five different varieties (*C. moschata* species: Lunga di Napoli, Moscata di Provenza, and Violina rugosa; *C. maxima* species: Hokkaido, and Mantovana) were harvested in November 2022 and 2023 in the Umbria region (Perugia, Central Italy, altitude 180 m a.s.l., coordinates 43°04′24″ N 12°33′04″ E). The peel was carefully removed with a sharp knife. The filaments and seeds were removed by hand, and the pulp was cut into cubes and preserved. Small pieces of peel were dried (40 °C) in a ventilated oven (Binder, Series ED, Tuttlingen, Germany) up to constant weight. The dried peels were crushed into a fine powder in a kitchen blender (Type KM28, Kenwood, Hampstead, UK) and then sieved through a stainless-steel sieve (Ø 200 µm, Controls, Milan, Italy). The fine powder was stored at room temperature in amber glass vials, away from light and humidity. An aliquot of the obtained powder (PPP) was subjected to color analysis, another aliquot was used for the extraction of carotenoids, and the following analyses (spectrophotometric and chromatographic). The main part was used to produce chicken burgers fortified with 4% PPP.

The (±)-6-hydroxy-2,5,7,8-tetramethylchromane-2-carboxylic acid (Trolox) and 2,2′-azino-bis(3-ethylbenzothiazoline-6-sulphonic acid) diammonium salt (ABTS) were from Sigma-Aldrich (Milan, Italy). Hexane and isopropanol were purchased from VWR (Milan, Italy). Lutein (≥92%) was from Extrasynthese (Genay, France), and β-carotene (>97.0%) was from TCI (Tokyo Chemical Industry) chemicals (Toshima, Tokyo, Japan). Zeaxanthin dipalmitate was small-scale isolated from a hydroalcoholic extract of goji berries using high-performance liquid chromatography with diode array detection (HPLC-DAD) and then used as a standard [17].

### 2.2. Color Analysis

A color analysis was carried out on PPP, the ingredient used to fortify the meat, as well as on the fresh minced meat mixture added with 4% PPP (before cooking). The analysis was performed using the EOPTIS CLM194 colorimeter (Metreo Solutions, Rome, Italy). The results (L*, a*, b* parameters) were expressed by the CIELAB scale. C* and H* parameters (chroma and hue angle, respectively) were calculated by the EasyRGB color calculator [18]. The color measurement of each sample was carried out in triplicate, and the average was calculated.

### 2.3. Extraction of Carotenoids and Determination of Total Carotenoids

The isolation of carotenoids of PPP was carried out (hexane: isopropanol 60:40 *v/v*, 30 min, 45 °C) by UAE using an ArgoLab sonication bath (mod. AU-65, Carpi, Italy). The total carotenoid content (TCC) was measured at 450 nm by a spectrophotometer (Lambda PerkinElmer, Inc., Waltham, MA, USA). The results were reported as µg β-carotene equivalents per gram of dry weight (µg β-CE/g DW) [17].

### 2.4. Determination of Antioxidant Activity

ABTS and oxygen radical absorbance capacity (ORAC) assays were used to evaluate the antioxidant activity of PPP extracts. The ABTS and ORAC methods were developed in previous works [17,19]. The results of the ABTS assay were reported as µg Trolox equivalents per gram of dry weight (µg TE/g DW). The results of the ORAC assay were expressed as Trolox equivalents (µg TE/g).

### 2.5. HPLC-DAD Analysis of Carotenoids

The qualitative and quantitative profiles of carotenoids were obtained by HPLC-DAD analysis performed using a Thermo Spectra Series pump (Thermo Scientific, Rockford, IL, USA) coupled with a UV 6000 LP DAD (Thermo Scientific, Waltham, MA, USA). A C30 Develosil column (250 × 4.6 mm i.d., 5 µm, Nomura Co., Kyoto, Japan) was used. The mobile phases were methanol:water (97:3 *v/v*) and methyl tert-butyl ether, respectively. The gradient HPLC-DAD method and the validation procedure were detailed in another paper [20]. Data acquisition was carried out by Excalibur software (version 1.1) (Chromatographic Specialties Inc., Brockville, ON, Canada). The calibration curves of standard solutions of β-carotene, lutein, and zeaxanthin dipalmitate were used for the quantification. Lutein was chosen for the quantification of non-esterified carotenoids (expressed as µg Lutein Equivalents/g, µg LE/g), being the most representative compound of this type of carotenoids, while zeaxanthin dipalmitate was chosen for the quantification of esterified carotenoids (expressed as µg Zeaxanthin Dipalmitate Equivalents/g, µg ZDE/g). The structural confirmation of the carotenoids was performed using liquid chromatography-quadrupole time-of-flight tandem mass spectrometry (LC-Q-TOF-MS/MS), with the experimental conditions detailed in another paper [20]. The identification of analytes was performed by comparing experimental spectra with those present in online MS/MS libraries (Human Metabolome Database or HMDB and MoNA MassBank of North America) and previous papers [21,22].

### 2.6. Production of Fortified Burger

Chicken burgers (100 g each) were hand-prepared according to the following recipe: chicken minced meat (100, 70, and 50%), beef minced meat (0, 30, and 50%, respectively), egg (1%), and salt (2%). The PPP, produced as reported in Section 2.1, was incorporated into minced meat at a 4% level to obtain fortified burgers. Before cooking, physical-chemical, microbiological, and color analyses were carried out on the minced meat mixture (raw meat) with 4% PPP added. Afterward, chicken burgers (four for each recipe) were cooked for 6 min (4 min on one side, and 3 min on the other) at 750 W in a domestic microwave with Tupperware^®^ MicroPro Grill equipment (Figure S1). They were then left to cool to room temperature for the successive sensory evaluation.

### 2.7. Physical-Chemical and Microbiological Analysis

An S40 SevenMulti digital pH-meter (Mettler-Toledo Italia, Novate Milanese, Italy) was used to measure the pH value of the fortified burgers. Burger meat, mixed with distilled water, was centrifuged at 2150× *g* for 5 min (Neya A 8–50). The water activity (aw) of burgers was measured with a dew-point HygroLab3 hygrometer (Rotronic, Hauppauge, NY, USA).

Regarding microbiological analysis, the samples were analyzed after the production (T0), and then after 24, 48, and 96 h (T24, T48, and T96, respectively) [23]. Samples were stored at refrigeration temperature (4 ± 2 °C) in the dark. In brief, for each analysis, meat was homogenized by using a stomacher in a sterile bag with peptone water (Oxoid, Basingstoke, UK). 10-fold dilutions were obtained using sterile tubes with 9 mL of Maximum Recovery Diluent (Oxoid, Hampshire, UK). Dilutions were inoculated in triplicate on different culture media. For each analysis, eight bacterial populations were evaluated: total aerobic mesophilic microbiota, *Lactobacillus* spp., *Lactococcus* spp., enterococci, *Staphylococcus* spp., Enterobacteriaceae, total coliforms, and *Pseudomonas* spp. Total aerobic mesophilic microbiota was determined on Plate Count Agar (PCA, Oxoid) at 30 °C for 72 h; *Lactococcus* spp. on M17 agar (Oxoid) 10% *v*/*v* lactose at 37 °C for 48 h; *Lactobacillus* spp. on Man, Rogosa, and Sharpe Agar (MRS, Oxoid) pH 5.5 at 30 °C for 72 h under anaerobic conditions (gas generating kit, Oxoid); enterococci on enterococcus agar (ENT, Oxoid) at 37 °C for 48 h, *Staphylococcus* spp. on Baird Parker agar (BP, Oxoid) containing Egg Yolk Tellurite (Oxoid) at 37 °C for 48 h, Enterobacteriaceae on violet red bile glucose agar (VRBG Oxoid) at 37 °C for 24 h, total coliforms on violet red bile lactose agar (VRBL, Oxoid) at 37 °C for 24 h, and *Pseudomonas* spp. on pseudomonas agar base (PS103, Oxoid) at 30 °C for 24 h. At the end, the number of colonies was converted to the log of colony-forming units per gram (log CFU/g), and the mean was calculated for each analysis. Sensitivity for spread plate was 102 CFU/g, and for pour plate was 10 CFU/g, and the 95% confidence limit was set between ±37% and ±12%. All plates with fewer than 30 CFU were excluded from data analysis. When this criterion was applied to the lowest dilution, results were recorded as <300 for the pour plate and <3000 for the spread plate [24].

### 2.8. Sensory Analysis

A sensory evaluation was conducted on chicken-based burgers containing 100%, 70%, and 50% chicken meat, prepared and cooked as previously described. A total of 22 semi-trained volunteer assessors, previously taking part in sensory evaluation techniques, were selected for the test. The training involved both theoretical and practical components, including an introduction to basic sensory principles, familiarization with the specific attributes to be evaluated (appearance, odor, flavor, and texture), and calibration sessions using reference samples to align perceptions across the panel. Practice sessions were held to improve the consistency and reproducibility of individual responses, and mock evaluations were carried out to ensure that panelists could correctly use the 7-point scales and understand the test protocols. The objectives of the evaluation were clearly explained to all participants. The panel included an equal number of male and female assessors, aged between 20 and 60 years. Participants were asked to evaluate the samples based on visual and olfactory attributes, flavor, and texture. Each 100 g burger was cut into six pieces and served on eco-friendly dishes. Samples were prepared out of the assessors’ view. The three fortified samples were presented in a random order on the same dish, with a blind code (three-digit numbers chosen randomly). This allowed us to label and keep track of samples without giving away any biased information. Subdued lighting was used to minimize visual bias. The assessors were also asked to report their general food preferences, specifically whether they regularly consume meat. Each assessor received evaluation forms with a 7-point intensity scale for each attribute (1 = minimum intensity, 7 = maximum intensity). The use of a 7-point scale was chosen as it provides sufficient sensitivity to detect differences among samples while maintaining ease of use for semi-trained panelists. The tastings took place in individual booths constructed according to ISO 4121:2003 guidelines [25]. Still water and unsalted bread were provided to cleanse the palate between samples. Evaluations were conducted under natural light at room temperature, in separate test sessions held at the same time of day (between 12:00 and 14:00). Overall product acceptability was rated using a 7-point structured hedonic scale (0 = hated it, 7 = loved it).

### 2.9. Statistical Analysis

All analytical determinations were reported as mean value ± standard deviation (SD) of three replicates. Statistical significance of pH, TCC, ABTS, ORAC, color, and chromatographic data was assessed using one-way analysis of variance (ANOVA), followed by Tukey’s HSD (honestly significant difference) post hoc test. A *p*-value less than 0.01 was considered statistically significant. GraphPad PRISM version 9.3.1 (GraphPad Software, Boston, MA, USA) and Excel Version 16.91 (Microsoft, Redmond, WA, USA) for Windows were used for statistical analysis and graph generation. Statistical analysis of aw and microbiological data was performed with GraphPad Prism, version 8.4.3 for Mac OS (GraphPad Software, Boston, MA, USA). Analysis of variance (ANOVA) followed by Tukey’s multiple comparisons test was performed considering the product type as the treatment. A *p*-value < 0.05 was considered to be significant.

## 3. Results and Discussion

### 3.1. Color Characteristics of PPP

Recently, the demand for natural antioxidants has increased. Research efforts are focused on identifying plant sources that could serve as sustainable alternatives for preserving the quality of meat and its derivatives [8]. In this research, the peel was recovered from pumpkins of five varieties harvested in the same company in the same harvesting period for two successive years (2022 vs. 2023). It is known that the nutritional composition of pumpkin peels is lower in carbohydrates and lipids than the pulp; moreover, it contains a high concentration of carotenoids, health-promoting phytochemicals responsible for the chromatic aspects [7]. Initially, the colorimetric analysis of PPP was performed. Table 1 shows the results of the CIELAB and CIELCH color parameters of peels of five pumpkin varieties (Hokkaido, Lunga di Napoli, Mantovana, Moscata di Provenza, and Violina rugosa), harvested in 2022 and 2023. The CIELAB color space is a three-dimensional color space defined by the International Commission on Illumination (CIE). It covers the entire gamut of human color perception and is based on the opponent model of human vision, where red and green form an opponent pair and blue and yellow form an opponent pair. The relative scale (L*a*b*: L*, lightness; a*, red-green value, and b*, yellow-blue value) is used to express colorimetric results. The lightness value, L*, defines black at 0 and white at 100. The a* axis relates to the green-red opponent colors, with negative values indicating green and positive values indicating red. The b* axis refers to the blue-yellow opponents, with negative numbers indicating blue and positive values indicating yellow. Regarding the CIELCH parameters, the C* value represents color saturation (quite simply, chroma describes the vividness or dullness of a color). Hue angle (H* value) represents the chromaticity or tone of color (quite simply, hue is how an object’s color is perceived). C* and H* values were calculated using a calculator by entering the values of a* and b* parameters.

Regarding pumpkins harvested in 2023, the L* values ranged from 61.73 for Mantovana to 74.44 for Violina rugosa. The lightness values were lower for pumpkins harvested in 2022 (56.79–72.43). For the redness (a* parameter), values ranged from −6.96 to 13.15 for Mantovana and Hokkaido, respectively. In 2022, no negative values were detected (0.37–15.01). In terms of yellowness (b* parameter), the pumpkins harvested in 2022 and 2023 showed the highest value for Hokkaido (56.96 and 69.13) and the lowest for Moscata di Provenza (31.42 and 28.35). In 2023, the C* values ranged from 28.75 to 70.37, while the H* values ranged from 79.23 to 97.18. This data indicated that three varieties (Hokkaido, Moscata di Provenza, and Violina rugosa) fell within the range of red to yellow hue (first quadrant of hue angle: 0–90°). In contrast, two varieties (Lunga di Napoli and Mantovana) were located in the yellow tending towards green (second quadrant of hue angle: 90–180°), as evidenced by their negative a* values. Statistically different results (*p* < 0.01) were obtained from the comparison between the color parameters of all varieties, harvested in both years (Table S1a–c and Figure S2a–c). A wide range of values of color parameters has been reported for pumpkin peel [20,26].

### 3.2. TCC and Antioxidant Activity of Carotenoid Extracts from PPP

**Figure 1:**
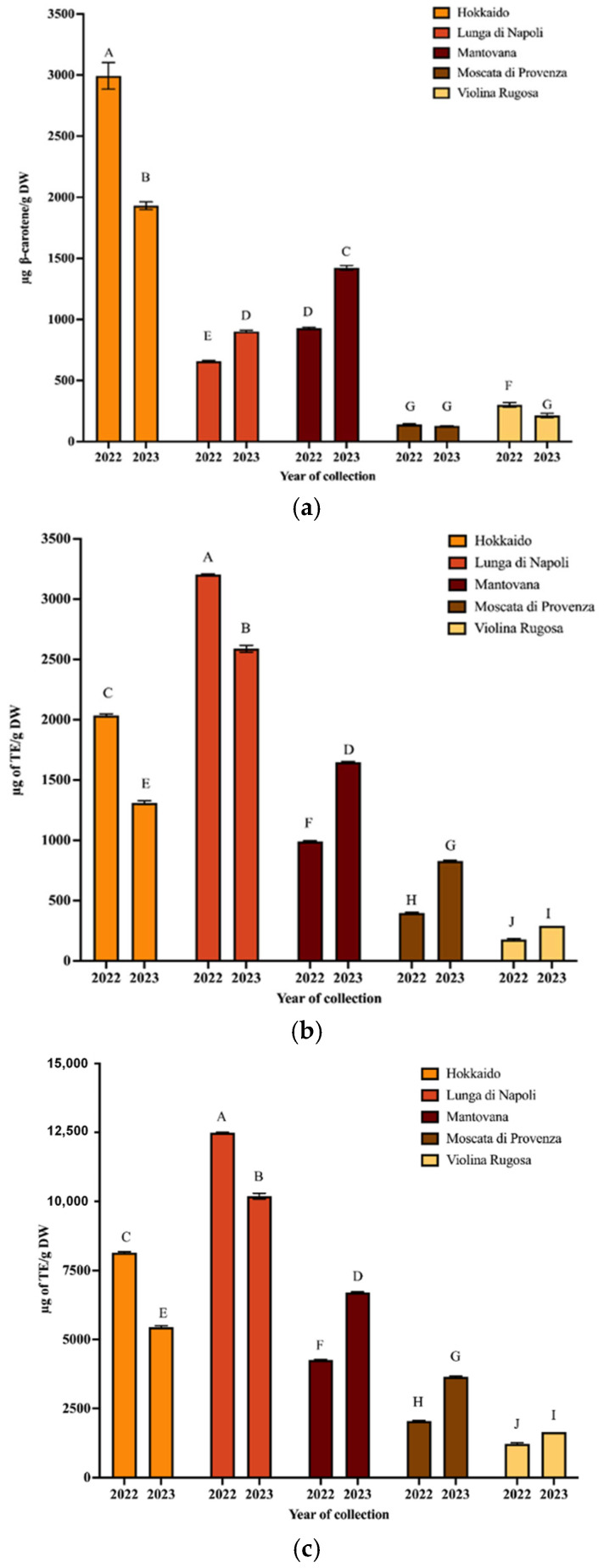
TCC (**a**), ABTS (**b**), and ORAC (**c**) values of carotenoid extracts from PPP obtained from pumpkin harvested in 2022 and 2023 (mean values ± SD, *n* = 3). Different letters indicate significant differ-ences with a *p*-value < 0.01.

To isolate the carotenoids of PPP, the UAE technique was applied using extraction conditions optimized in a previous paper [17]. Then, the extracts were spectrophotometrically analyzed to obtain the values of TCC and antioxidant activity (Figure 1a–c).

As regards TCC values, it can be observed that the Hokkaido variety provided the highest values for both years, while the Moscata di Provenza variety provided the lowest. Regarding the total antioxidant capacity, the ABTS values of peel extracts ranged from 175.6 to 13,204.46 µg TE/g for Violina rugosa and Lunga di Napoli, respectively (2022). In 2023, the same varieties exhibited the lowest and highest values, respectively. The ORAC values confirm the trend of ABTS values (R^2^ ≥ 0.9990) for both collection years. Hokkaido peel had an ABTS value of 2036.03 µg TE/g (ORAC 8141.25 µg TE/g) in 2022 and 1311.47 µg TE/g (ORAC 5447.37 µg TE/g) in 2023. These data confirmed that the composition of pumpkin and its waste can vary widely depending on variety, collection time, and climatic conditions [27]. A wide range of variability of antioxidant activity and carotenoid composition was found in a previous study where eight pumpkin varieties were analyzed [20]. It should be emphasized that in this work, to minimize variability, pumpkins from the same farm (grown in the same geographic area) were collected. Confirming that environmental conditions modify the chemical composition, some data on the weather conditions of the harvesting pumpkin zone were detailed in the following. The temperature between April and August was lower in 2023 than in 2022, while the total rainfall was higher [28]. These conditions seem to decrease carotenoid synthesis in some varieties, such as Hokkaido, while improving it for the Mantovana variety. Finally, statistically different results (*p* < 0.01) were obtained from the comparison between the spectrophotometric parameters of all varieties harvested in both years (Table S2a–c).

### 3.3. Chromatographic Characterization of Carotenoid Extracts from PPP Extract

**Figure A1:**
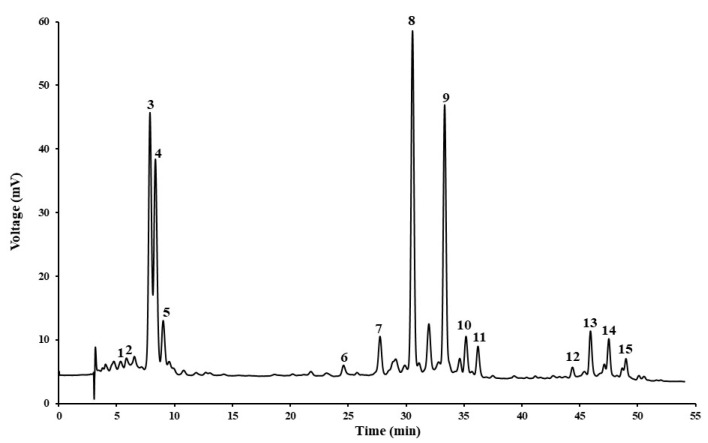
HPLC-DAD chromatographic profile of Hokkaido peel (1. Neoxanthin; 2, violaxanthin; 3, antheraxanthin; 4, lutein; 5, zeaxanthin; 6, violaxanthin myristate; 7, lutein palmitate; 8, antheraxanthin myristate; 9, antheraxanthin palmitate; 10, β-carotene; 11, violaxanthin dimyristate; 12, lutein laurate myristate; 13, lutein dimyristate; 14, lutein myristate palmitate; 15, lutein dipalmitate).

The carotenoids of PPP were analyzed using the HPLC-DAD method. Figure A1 (Appendix A) shows the chromatographic profile of the carotenoids of Hokkaido peel. The relative values of linearity, accuracy, precision, and the validation procedure were reported in previous papers [17,20]. Table 2 shows the contents of carotenoids of the five pumpkin varieties (2022 and 2023), considering free, mono- and di-esterified xanthophylls. Generally, it can be affirmed that free xanthophylls were always less abundant than esterified ones, considering all varieties and both vintages. The content of free xanthophylls ranged from 1.75 µLE/g to 621.93 µLE/g in 2022, and from 2.54 µLE/g to 367.97 µLE/g in 2023 (Moscata di Provenza always showed the lowest value, while Hokkaido always showed the highest). Among free xanthophylls, neoxanthin and zeaxanthin were not detected in Lunga di Napoli and Moscata di Provenza. For both harvesting years, Moscata di Provenza was the variety with the lowest content of carotenoids (both free and esterified).

Regarding the esterified xanthophylls, the mono-esterified compounds include violaxanthin and antheraxanthin myristate, lutein and antheraxanthin palmitate. Among the di-esterified forms, violaxanthin dimyristate, antheraxanthin dilaurate, lutein laurate myristate, lutein dimyristate, lutein myristate palmitate, and lutein dipalmitate were also detected. In 2023, three varieties (Hokkaido, Mantovana, and Moscata di Provenza) had the mono-esterified more abundant than the di-esterified, while in 2022, Hokkaido showed a higher value for di-esterified. For the Hokkaido variety, it can be noted that the lutein was the main carotenoid esterified with lauric (C12:0), myristic (C14:0), or palmitic (C16:0) acids, in mixed (laurate, myristate, palmitate) or homogeneous (myristate and palmitate) forms. Carotenoids, like lutein, zeaxanthin, and β-carotene, have distinct effects on human health, including vision, immune function, and cardiovascular health [6]. Statistically different results (*p* < 0.01) were obtained from the comparison between the content of mono-esterified compounds of the same pumpkin variety (Hokkaido, Mantovana) of two harvesting years and the di-esterified compounds. Other interesting differences were reported in Table S3a–d and Figure S3a–d.

In other papers, pulp and by-products (filaments and peel) of the Hokkaido variety were analyzed [17,20]. Some authors reported only five carotenoids identified in the mesocarp of Hokkaido pumpkin, including antheraxanthin, β-carotene, lutein, violaxanthin, and neoxanthin (17.50, 15.04, 10.14, 5.76, and 4.95 μg carotenoid/g pumpkin fresh weight, respectively) [29]. Good correlations for both years (R^2^ ≥ 0.8845) were found between TCC and total carotenoids determined by HPLC-DAD.

### 3.4. Nutritional Considerations of Meat Used for Burgers

PPP can be considered a valuable and sustainable functional ingredient, providing an excellent and cost-effective source of carotenoids and fiber. After analyzing the various pumpkin samples, the Hokkaido variety was selected for the preparation of chicken burgers. It had the highest nutrient content in both years and displayed a distinctive chromatographic profile.

Table 3 shows the values of nutritional labels of meat used to produce burgers, as reported on the packaging of beef and chicken minced meats (g/100 g). These data, providing the energy value and the amounts of fat, saturated, carbohydrates, sugars, protein, and salt in the food, have been mandatory since December 2016, based on Regulation (EU) No. 1169/2011 [30]. It can be noted that chicken (breast) meat showed a lower energy value than beef, due to the lower content of total fat (4.9 vs. 22.8 g/100 g). Moreover, it is characterized by lower saturated fatty acids (1.5% vs. 11.1%) and higher protein content (21 vs. 17.3 g/100 g).

Numerous data sources indicate that chicken meat consumption is now the preferred (less expensive) animal protein choice in the EU, according to a recent US Department of Agriculture (USDA) Global Agricultural Information Network (GAIN) report (USDA-GAIN, 2024) [31]. After pork, it is the second-largest meat consumed in the EU. In several EU countries, such as Italy, Germany, France, and Poland, the shift to chicken meat consumption is also aided by the perception that it is a healthier and leaner meat, and it is generally easier (low lipid content) to prepare and economical. Chicken meat is recommended for those looking to lower their fat intake and for individuals with heart and coronary diseases. Recently, the interesting nutritional characteristics of chicken meat were implemented with functional ingredients of vegetable origin to obtain value-added products [32].

### 3.5. Physical-Chemical Analysis and Color of Fortified Chicken Burgers

At the beginning of the experiment, the pH values of the minced meat mixture decreased slightly with the increase of the percentage of beef meat (6.13 ± 0.06, 6.03 ± 0.06, and 5.98 ± 0.04 for 100, 70, and 50% chicken burger, respectively). Similar pH values have been reported for burgers prepared with chicken meat and pork backfat with added pumpkin seeds (i.e., 6.16 and 6.20 at 1 and 2%, respectively) [9].

The evaluation of the color of chicken burgers is important because it is a characteristic capable of influencing consumption attitudes, and the relative results before and after the addition of 4% PPP were reported in Table 4. The b* and C* values decreased with the increase in the percentage of beef meat.

It can be noted that the L* values were always higher (*p* < 0.01) for minced meat without PPP, while a*, b*, C*, and H* were always lower (*p* < 0.01) than fortified burgers. These results could be related to the presence of PPP from Hokkaido (orange color), which showed high values of a*, b*, and C*. Moreover, this trend could be associated with browning reactions, such as the Maillard reaction or oxidative phenomena, shifting the product color towards a reddish or brownish hue, with less color lightness (L*). Table S4a–c and Figure S4a–c reported statistically different results (*p* < 0.01) obtained from the comparison among the color parameters of burgers prepared with 100, 70, and 50% chicken meat, with and without PPP. A different trend of color parameters was observed for pumpkin flowers (fresh, dried, frozen) in chicken patties [33].

A_w_ values ranged from 0.989 (T0) to 0.956 (T96) in 100% chicken burgers, from 0.992 to 0.973 in 70%, and from 0.996 to 0.987 in 50% (Table 5). Statistically significant (*p*-value < 0.01) differences were observed at T48 and T96 for 50% chicken burgers when compared to 100%.

### 3.6. Microbiological Characteristics of Fortified Chicken Burgers

**Figure 2:**
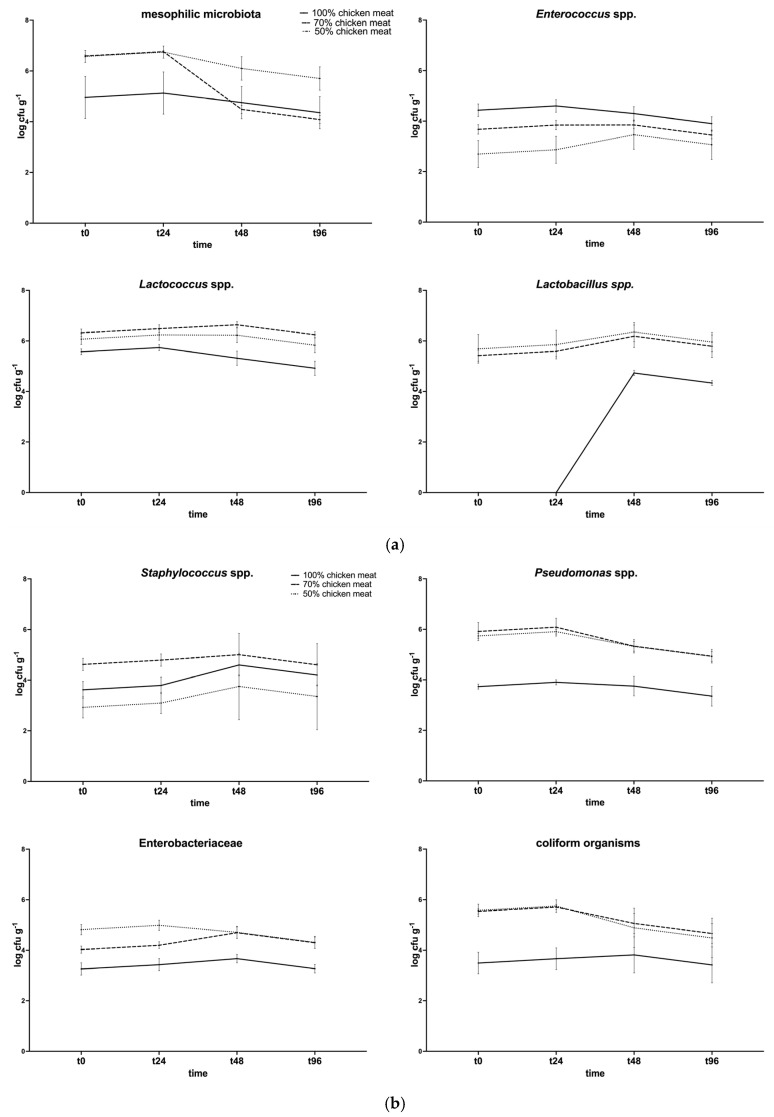
Microbiological analysis of chicken burgers, prepared with 100, 70, and 50% chicken meat and 4% PPP. (**a**) Total aerobic mesophilic flora, *Enterococcus* spp., *Lactococcus* spp., and *Lactobacillus* spp.; (**b**) *Staphylococcus* spp., *Pseudomonas* spp., *Enterobacteriaceae*, and total coliforms.

The microbiological quality of the raw samples was certified before proceeding with the sensory analysis. Figure 2a,b shows the microbiological analysis results.

Statistically significant differences (*p* < 0.05) were observed for total aerobic mesophilic flora at T48 and T96 (70% vs. 50%); for *Lactobacillus* spp. and *Lactococcus* spp. (100% vs. 70% and 50%); for enterococci at T0 and T24 (100% vs. 70% and 40%). The bacterial load of these groups at the beginning of the process was approximately 10^5^ CFU/g, while, at the end of ripening, it decreased to 10^4^ CFU/g. On average, these bacterial populations reached higher values in batches with bovine meat. As for spoilage bacterial populations, statistically significant differences were observed for *Staphylococcus* spp. at T0 and T24, when the 70% group showed higher levels. For *Enterobacteriaceae*, coliforms, and *Pseudomonas* spp., the 100% group always had a lower level when compared to the 70% and 50% groups. *Staphylococcus aureus* was not detected during the experiment.

Previous studies determined the microbiological characteristics of new functional foods enriched with bioactives derived from food waste, including apple pomace [34,35]. Similarly, Zargar et al. studied the effect of refrigerated storage on aerobically packaged cooked chicken sausages, highlighting that coliforms were not detected throughout the storage period [36]. These studies underscore the importance of microbiological analysis in ensuring the safety and quality of food products. Apple pomace, rich in phenolic compounds and dietary fibers, not only enhances the nutritional profile of fortified foods but also exhibits antimicrobial properties that can contribute to a more stable microbiological environment, potentially extending product shelf life [37,38]. These studies demonstrated that adding apple pomace to meat products can affect their microbiological characteristics. Freeze-dried apple pomace in turkey sausages showed antimicrobial effects, which reduced microbial loads during storage [39]. Similarly, while specific microbiological changes were not detailed in salami fortified with apple pomace, the antioxidant activity of apple pomace could contribute to microbial stability [34].

### 3.7. Sensory Evaluation of Fortified Chicken Burgers

Initially, the assessors evaluated the palatability and color of burgers prepared with 2, 4, and 8% PPP derived from the five pumpkin varieties. The best sensorial acceptance was reported for burgers added with 4% PPP derived from Hokkaido peel (orange/red skin color), while the ones with 8% were too sweet.

**Figure 3:**
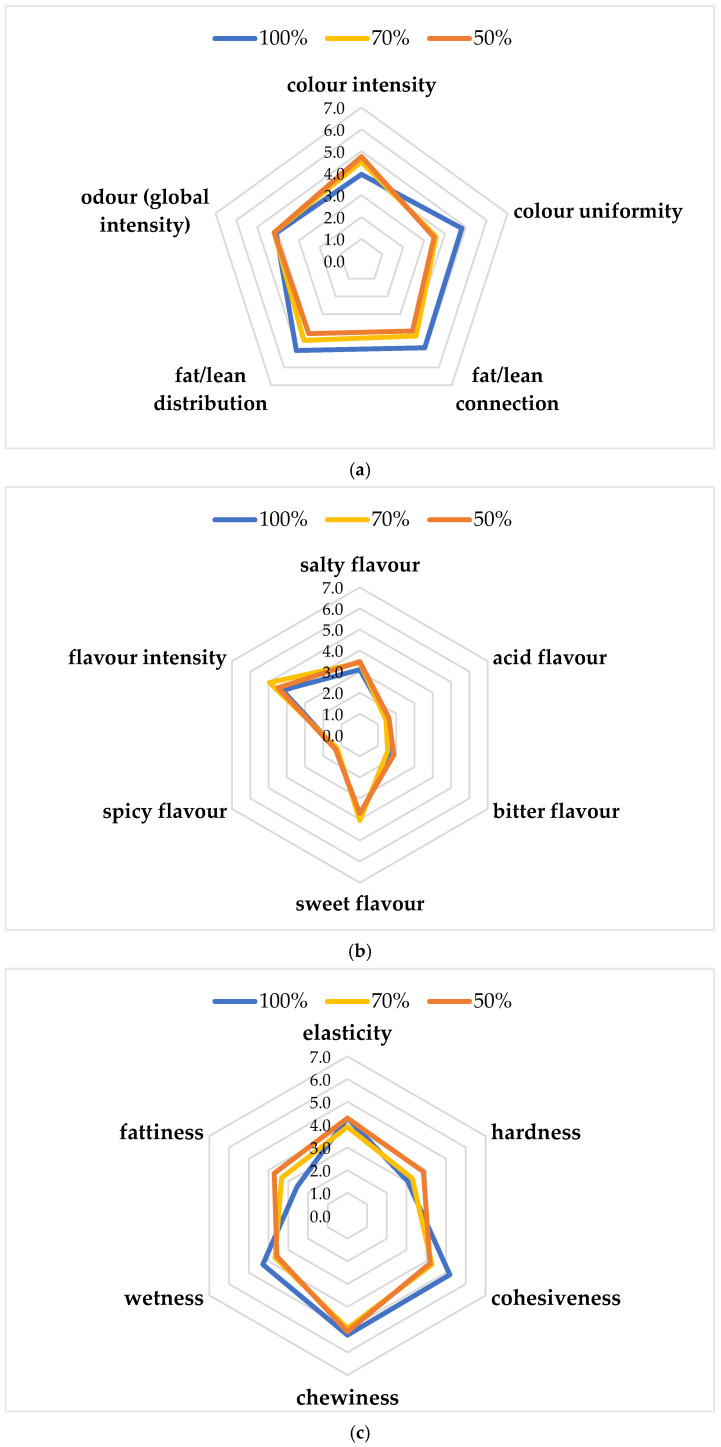
Sensory descriptive analysis: (**a**) Appearance attributes and odor; (**b**) Basic tastes; (**c**) Texture attributes.

Figure 3a–c shows the results of the sensory evaluation of the functional burgers. Regarding appearance attributes (Figure 3a), similar scores (4.76–4.52) were observed for the color intensity of chicken burgers with 50 and 70% chicken meat, while the lowest value (3.95) was obtained for the 100% chicken burger. The global odor intensity did not show differences among the three products, while color uniformity, fat/lean connection, and distribution showed the highest values for the 100% chicken burger. As regards basic tastes (Figure 3b), all samples obtained similar scores for sweet, spicy, and acid flavor. Flavor intensity was higher for 70% (4.95), followed by 50% (4.48) and 100% (4.24) burger. Salty flavor was higher for 50% (3.48), followed by 70% (3.43) and 100% (3.10). The highest bitter flavor was reported for the 50% burger. As regards texture attributes (Figure 3c), elasticity, cohesiveness, chewiness, and wetness were the highest for the 100% chicken burger (4.29, 5.19, 5.24, and 4.29, respectively), while hardness and fattiness were the highest for the 50% chicken burger (3.86 and 3.71, respectively). The highest score for overall acceptability was achieved by the 70% chicken burger (5.95 ± 1.02), followed by the 100% (5.29 ± 1.06) and 50% (4.86 ± 1.39). In particular, the 70% chicken burger showed the highest score for sweet flavor and flavor intensity. This result could be because lipids are responsible for the desirable sensory characteristics of meat and meat products, providing flavor and contributing to their juiciness, making the product more acceptable. The sensory quality of chicken meat nuggets with different levels of powdered pumpkin and chia seeds was evaluated after refrigerated storage under aerobic conditions [40]. In this study, the scores for color decreased with the advancement in the storage period, which is irrespective of the treatments. Generally, all treatments had color scores around 7.0, which means very good acceptability on the 12th day of storage. Similarly, other authors studied the sensory attributes of aerobically packaged chicken sausages added with 12% of mature pumpkin, showing a descending trend with an increase in storage (21 days) [36]. The authors reported that the sensory attributes and overall acceptability were significantly affected during 21 days of refrigerated storage, following a descending trend with increase in storage days.

## 4. Conclusions

The recent strategy based on animal food fortification with vegetable ingredients from agri-food waste fully fits into the circular economy action plan approved in Europe. Initially, an in-depth analytical characterization of the functional ingredient was performed. The results of this first part of the study highlighted that color characteristics, TCC, antioxidant activity, and carotenoid profile were highly variable and dependent on the pumpkin variety and harvesting year, even if the harvest area was the same. Afterward, Hokkaido peel powder, which exhibited interesting color, composition characteristics, and biological activity, was chosen as a bioactive ingredient to fortify (4%) burgers made with different percentages of chicken meat (100, 70, and 50%). All the fortified burgers were analyzed, and, based on sensory properties, the product with 70% chicken meat was found to be the best in terms of acceptability, even if a larger sampling is desirable. This by-product of pumpkin processing improves the functional properties of the chicken burgers and contributes to promoting sustainable commodities. The evaluation of bioaccessibility and bioavailability, as well as in vivo studies, will be necessary to evaluate the real benefits to consumer health. This paper highlights that pumpkin peel represents an underutilized resource with promising applications to address global nutritional challenges, even if a deep investigation into economic feasibility is needed. Nevertheless, in the future, different meals added with PPP could be formulated for a long-term stability study to evaluate their shelf life and consumer behavior. Trials under real-life storage conditions or market studies to assess consumer interest could be useful for the progress of the work.

## Figures and Tables

**Table 1:** Values of color characteristics of PPP (mean values ± SD, *n* = 3).

Color Parameters	Hokkaido	Lunga di Napoli	Mantovana	Moscata di Provenza	Violina Rugosa
2022					
L*	58.66 ± 0.02	56.79 ± 0.01	59.53 ± 0.01	66.02 ± 0.02	72.43 ± 0.02
a*	15.01 ± 0.01	0.37 ± 0.02	0.38 ± 0.01	2.59 ± 0.01	0.87 ± 0.02
b*	56.96 ± 0.02	35.80 ± 0.03	36.98 ± 0.02	31.42 ± 0.03	33.94 ± 0.04
C*	58.90 ± 0.01	35.80 ± 0.02	36.98 ± 0.02	31.53 ± 0.02	33.95 ± 0.01
H*	75.24 ± 0.02	89.41 ± 0.01	89.41 ± 0.01	85.29 ± 0.02	88.53 ± 0.03
2023					
L*	64.27 ± 0.01	72.05 ± 0.01	61.73 ± 0.01	66.58 ± 0.01	74.44 ± 0.01
a*	13.15 ± 0.01	−4.78 ± 0.03	−6.96 ± 0.02	4.81 ± 0.01	0.73 ± 0.01
b*	69.13 ± 0.02	37.94 ± 0.04	55.47 ± 0.02	28.35 ± 0.03	39.15 ± 0.03
C*	70.37 ± 0.01	38.24 ± 0.02	55.90 ± 0.01	28.75 ± 0.00	39.16 ± 0.01
H*	79.23 ± 0.01	97.18 ± 0.01	97.15 ± 0.01	80.37 ± 0.02	88.93 ± 0.02

L*, color lightness; a*, degree of greenness (negative)-redness (positive); b*, degree of blueness (negative)-yellowness (positive); C*, chroma; H*, hue angle.

**Table 2:** Contents of carotenoids of PPP (mean values ± SD, *n* = 3).

Carotenoids	Hokkaido	Lunga di Napoli	Mantovana	Moscata di Provenza	Violina Rugosa
2022					
**Free xanthophylls** (µg LE/g)	621.93 ± 14.96	100.78 ± 0.34	223.41 ± 2.24	1.75 ± 0.06	3.51 ± 0.18
neoxanthin	9.40 ± 0.18	-	8.83 ± 0.01	-	-
violaxanthin	6.89 ± 0.01	16.57 ± 0.13	9.20 ± 0.15	-	0.63 ± 0.00
antheraxanthin	312.94 ± 6.49		36.64 ± 0.13	1.05 ± 0.04	-
lutein	216.53 ± 6.77	84.22 ± 0.47	147.55 ± 1.78	0.70 ± 0.02	2.88 ± 0.17
zeaxanthin	65.15 ± 1.18		21.18 ± 0.44	-	-
**Mono-esterified xanthophylls** (µg ZDE/g)	18,870.52 ± 431.92	925.27 ± 21.46	2032.76 ± 14.16	123.32 ± 6.58	230.49 ± 13.56
violaxanthin myristate	261.35 ± 7.78		88.67 ± 2.32	-	38.02 ± 3.23
lutein palmitate	1227.50 ± 4.45	276.15 ± 19.09	435.02 ± 3.01	13.03 ± 0.49	27.37 ± 2.16
antheraxanthin myristate	9972.09 ± 209.03	186.23 ± 3.36	868.06 ± 6.59	-	-
antheraxanthin palmitate	7409.59 ± 219.56	462.88 ± 5.74	641.01 ± 26.08	110.28 ± 6.09	165.11 ± 8.18
**Di-esterified xanthophylls** (µg ZDE/g)	2297.61 ± 65.41	986.86 ± 12.72	395.45 ± 5.98	34.06 ± 0.53	299.98 ± 0.91
violaxanthin dimyristate	94.61 ± 2.76	-	-	6.17 ± 0.30	-
antheraxanthin dilaurate	-	-	-	-	-
lutein laurate myristate	303.23 ± 5.47	293.03 ± 1.00	58.41 ± 3.82	17.48 ± 0.64	163.75 ± 8.01
2023					
**Free xanthophylls** (µg LE/g)	367.97 ± 5.11	93.51 ± 2.08	202.35 ± 15.80	2.54 ± 0.06	3.87 ± 0.14
neoxanthin	11.03 ± 0.31	-	8.08 ± 0.22	-	0.93 ± 0.02
violaxanthin	8.95 ± 0.19	19.11 ± 0.03	14.12 ± 0.55	-	0.99 ± 0.03
antheraxanthin	154.90 ± 4.25	-	21.78 ± 1.12	1.49 ± 0.05	1.94 ± 0.14
lutein	134.45 ± 0.24	74.40 ± 2.05	139.80 ± 12.23	1.05 ± 0.01	-
zeaxanthin	49.26 ± 0.51	-	18.56 ± 1.68	-	-
**Mono-esterified xanthophylls** (µg ZDE/g)	9065.35 ± 208.35	652.18 ± 10.75	3968.30 ± 82.14	107.12 ± 9.00	166.88 ± 3.82
violaxanthin myristate	186.68 ± 4.45	-	255.51 ± 12.38	-	34.51 ± 0.09
lutein palmitate	666.36 ± 12.06	-	428.94 ± 2.05	19.28 ± 0.26	16.87 ± 0.18
antheraxanthin myristate	4635.53 ± 105.76	109.99 ± 3.56	1913.78 ± 153.76	-	-
antheraxanthin palmitate	3576.78 ± 86.09	542.18 ± 7.19	1370.08 ± 86.05	87.83 ± 9.26	115.50 ± 4.09
**Di-esterified xanthophylls** (µg ZDE/g)	1832.74 ± 29.80	937.12 ± 8.55	1079.37 ± 66.37	56.93 ± 3.21	294.17 ± 1.01
violaxanthin dimyristate	445.37 ± 13.30	74.02 ± 2.28	85.98 ± 0.57	15.52 ± 0.60	23.51 ± 0.09
antheraxanthin dilaurate	-	-	-	-	33.84 ± 0.52
lutein laurate myristate	174.95 ± 5.89	317.80 ± 0.54	97.27 ± 5.21	24.57 ± 2.09	129.09 ± 1.40

LE, lutein equivalents; ZDE, zeaxanthin dipalmitate equivalents; -, not detected.

**Table 3:** Nutritional label (data refers to 100 g) of minced meats used to produce burgers.

Meat	Energy Kcal (kJ)	Protein g	Carbohydrates (Sugars) g	Fat (Saturated) g	Fiber g	Salt g
Chicken	128 (538)	21	<0.5	4.9 (1.5)	0	0.35
Beef	275 (1138)	17.3	0	22.8 (11.1)	0	1.6

**Table 4:** Values of color characteristics of chicken burgers, prepared with 100, 70, and 50% chicken meat, without and with 4% PPP (mean values ± SD, *n* = 3).

	100%		70%		50%	
Color Parameters	Without PPP	With 4% PPP	Without PPP	With 4% PPP	Without PPP	With 4% PPP
L*	52.73 ± 0.02	45.22 ± 0.02	46.18 ± 0.01	39.35 ± 0.01	43.41 ± 0.02	41.51 ± 0.01
a*	11.81 ± 0.03	14.17 ± 0.02	18.48 ± 0.02	22.48 ± 0.02	20.12 ± 0.03	21.62 ± 0.02
b*	17.19 ± 0.04	25.29 ± 0.03	14.73 ± 0.04	32.60 ± 0.02	11.40 ± 0.01	34.29 ± 0.01
C*	20.86 ± 0.01	28.99 ± 0.01	23.63 ± 0.02	39.60 ± 0.01	23.13 ± 0.02	40.54 ± 0.02
H*	55.51 ± 0.03	60.74 ± 0.03	38.56 ± 0.01	55.41 ± 0.02	29.54 ± 0.01	57.77 ± 0.01

L*, color lightness; a*, degree of greenness (negative)-redness (positive); b*, degree of blueness (negative)-yellowness (positive); C*, chroma; H*, hue angle.

**Table 5:** Values of a_w_ of burgers prepared with 100, 70, and 50% chicken meat and 4% PPP, sampled at different times (mean values ± SD, *n* = 3).

	100%	70%	50%
T0	0.989 ± 0.003	0.992 ± 0.002	0.996 ± 0.003
T24	0.981 ± 0.004	0.989 ± 0.005	0.993 ± 0.001
T48	0.967 ± 0.002	0.987 ± 0.005	0.990 ± 0.001
T96	0.956 ± 0.002	0.973 ± 0.002	0.987 ± 0.002

## Data Availability

Data is contained within the article and Supplementary Materials.

## Supplementary Materials

The following supporting information can be downloaded at: https://www.mdpi.com/article/10.3390/antiox14060648/s1, Figure S1: Cooking equipment (a), chicken burgers before and after cooking (b) with different percentages of chicken meat: 100, 70, and 50%; Figure S2: Values of L* parameter (a), a* parameter (b), and b* parameter (c) of carotenoid extracts from PPP obtained from pumpkin harvested in 2022 and 2023 (mean values ± SD, *n* = 3). Different letters indicate significant differences with *p*-value < 0.01. LN; Figure S3: Contents of free xanthophylls (a; µg LE/g), mono-esterified (b; µg ZDE/g), and di-esterified (c; µg ZDE/g) xanthophylls, and β-carotene (d; µg/g) of extracts from PPP obtained from pumpkin harvested in 2022 and 2023 (mean values ± SD, *n* = 3). Different letters indicate significant differences with *p*-value < 0.01; Figure S4: Values (mean values ± SD, *n* = 3) of L* parameter (a), a* parameter (b), and b* parameter (c) of chicken burgers made with 100, 70, and 50% chicken meat, without (control) and with 4% PPP. Different letters indicate significant differences with *p*-value < 0.01; Table S1: Statistical analysis carried out on L* parameter (a), a* parameter (b), and b* parameter (c) of PPP (Tukey’s multiple comparisons test) of different pumpkin variety and harvesting years (2022–2023); Table S2: Statistical analysis carried out on TCC (a), ABTS (b), and ORAC (c) of PPP (Tukey’s multiple comparisons test) of different pumpkin variety and harvesting years (2022–2023); Table S3: Statistical analysis (Tukey’s multiple comparisons test) carried out on HPLC data of free xanthophylls (a), mono-esterified xanthophylls (b), di-esterified xanthophylls (c), and β-carotene (d) of different pumpkin variety and harvesting years (2022–2023); Table S4: Statistical analysis carried out on L* parameter (a), a* parameter (b), and b* parameter (c) of chicken burgers (Tukey’s multiple comparisons test) characteristics of chicken burgers, prepared with 100, 70, and 50% chicken meat, without (control) and with 4% PPP.

## Abbreviations

The following abbreviations are used in this manuscript:

PPP	peel powder of pumpkins
LE	lutein equivalents
ZDE	zeaxanthin dipalmitate equivalents
HPLC	high-performance liquid chromatography
DAD	diode array detector
UAE	ultrasound-assisted extraction
TCC	total carotenoid content
ORAC	oxygen radical absorbance capacity

## References

[1] (2025). EUROSTAT. https://ec.europa.eu/eurostat/statistics-explained/index.php?title=Food_waste_and_food_waste_prevention_-_estimates.

[2] Singh K., Kumar T., Patel V., Kumar V., Sharma S., Pradesh Krishi Vishvavidyalaya H., Pradesh H., Jyoti Rani I., Kavindra Singh C., Rani J. (2019). A review on the conversion of food wastes and by-products into value added products. Int. J. Chem. Stud..

[3] Faustino M., Veiga M., Sousa P., Costa E.M., Silva S., Pintado M. (2019). Agro-food byproducts as a new source of natural food additives. Molecules.

[4] Khalid W., Arshad M.S., Ranjha M.M.A.N., Rozanska M.B., Irfan S., Shafique B., Rahim M.A., Khalid M.Z., Abdi G., Kowalczewski P.Ł. (2022). Functional constituents of plant-based foods boost immunity against acute and chronic disorders. Open Life Sci..

[5] Aziz A., Noreen S., Khalid W., Ejaz A., Faiz ul Rasool I., Maham N., Munir A., Farwa N., Javed M., Ercisli S. (2023). Pumpkin and pumpkin byproducts: Phytochemical constitutes, food application and health benefits. ACS Omega.

[6] Hussain A., Kausar T., Sehar S., Sarwar A., Ashraf A.H., Jamil M.A., Noreen S., Rafique A., Iftikhar K., Quddoos M.Y. (2022). A comprehensive review of functional ingredients, especially bioactive compounds present in pumpkin peel, flesh and seeds, and their health benefits. Food Chem. Adv..

[7] Gavril Raţu R.N., Stoica F., Lips F.D., Constantin O.E., Stănciuc N., Aprodu I., Râpeanu G. (2024). Pumpkin and pumpkin by-products: A comprehensive overview of phytochemicals; extraction; health benefits; and food applications. Foods.

[8] Espinales C., Baldeón M., Bravo C., Toledo H., Carballo J., Romero-Peña M., Cáceres P.J. (2024). Strategies for healthier meat foods: An overview. Prev. Nutr. Food Sci..

[9] Saini R.K., Prasad P., Lokesh V., Shang X., Shin J., Keum Y.S., Lee J.H. (2022). Carotenoids: Dietary sources; extraction; encapsulation; bioavailability; and health benefits-a review of recent advancements. Antioxidants.

[10] Verma A.K., Banerjee R., Sharma B.D. (2015). Quality characteristics of low fat chicken nuggets: Effect of salt substitute blend and pea hull flour. J. Food Sci. Technol..

[11] Longato E., Lucas-González R., Peiretti P.G., Meineri G., Pérez-Alvarez J.A., Viuda-Martos M., Fernández-López J. (2017). The effect of natural ingredients (amaranth and pumpkin seeds) on the quality properties of chicken burgers. Food Bioproc. Technol..

[12] Serdaroğlu M., Kavuşan H.S., Îpek G., Öztürk B. (2018). Evaluation of the quality of beef patties formulated with dried pumpkin pulp and seed. Korean J. Food Sci. Anim. Resour..

[13] Rolim de Melo F.A.B., Fonseca Galvão M.B., Félix da Costa A., da Silva C.F., Campos Guerra J.M., Montenegro Stamford T.C. (2024). Development and evaluation of nutritional and quality standard of beef burger supplemented with pumpkin (*Cucurbita moschata*) seed flour. Foods.

[14] Öztürk T., Turhan S. (2020). Physicochemical properties of pumpkin (*Cucurbita pepo* L.) seed kernel flour and its utilization in beef meatballs as a fat replacer and functional ingredient. J. Food Process. Preserv..

[15] Haddad M.A., Al–Dalain S.Y., Al-Fraihat A.H., Parisi S., Parisi C., Arabiat S., Alqaraleh S.Y. (2023). Use of fresh pumpkin fruits for producing chicken sausage suggests functional properties. Curr. Res. Nutr. Food Sci..

[16] Abilmazhinova B., Rebezov M., Fedoseeva N., Belookov A., Belookova O., Mironova I., Nigmatyanov A., Gizatova N. (2020). Study chemical and vitamin composition of horsemeat cutlets with addition of pumpkin. Int. J. Psychosoc. Rehabil..

[17] Pinna N., Ianni F., Blasi F., Stefani A., Codini M., Sabatini S., Schoubben A., Cossignani L. (2022). Unconventional extraction of total non-polar carotenoids from pumpkin pulp and their nanoencapsulation. Molecules.

[18] EasyRGB (2025). Convert Color Data Into Different Standards and Color Spaces. https://www.easyrgb.com/en/convert.php.

[19] Persichetti E., De Michele A., Codini M., Traina G. (2014). Antioxidative capacity of Lactobacillus fermentum LF31 evaluated in vitro by oxygen radical absorbance capacity assay. Nutrition.

[20] Pinna N., Ianni F., Selvaggini R., Urbani S., Codini M., Grispoldi L., Cenci-Goga B.T., Cossignani L., Blasi F. (2023). Valorization of pumpkin byproducts: Antioxidant activity and carotenoid characterization of extracts from peel and filaments. Foods.

[21] Kurz C., Carle R., Schieber A. (2008). HPLC-DAD-MSn characterisation of carotenoids from apricots and pumpkins for the evaluation of fruit product authenticity. Food Chem..

[22] Petry F.C., Mercadante A.Z. (2016). Composition by LC-MS/MS of new carotenoid esters in mango and citrus. J. Agric. Food Chem..

[23] Grispoldi L., Karama M., Sechi P., Iulietto M.F., Hadjicharalambous C., Cenci-Goga B.T. (2021). Evaluation of a nitrite-free commercial preparation in the production of swine and roe deer (*Capreolus capreolus*) salami. Ital. J. Anim. Sci..

[24] Cenci-Goga B.T., Sechi P., Iulietto M.F., Amirjalali S., Barbera S., Karama M., Aly S.S., Grispoldi L. (2020). Characterization and growth under different storage temperatures of ropy slime-producing Leuconostoc mesenteroides isolated from cooked meat products. J. Food Prot..

[25] (2003). Sensory Analysis-Guidelines for the Use of Quantitative Response Scales.

[26] Sharma M., Bhat R. (2021). Extraction of carotenoids from pumpkin peel and pulp: Comparison between innovative green extraction technologies (ultrasonic and microwave-assisted extractions using corn oil). Foods.

[27] Kostecka-Gugała A., Kruczek M., Ledwózyw-Smolén I., Kaszycki P. (2020). Antioxidants and health-beneficial nutrients in fruits of eighteen Cucurbita cultivars: Analysis of diversity and dietary implications. Molecules.

[28] Regione Umbria; 2022; 2023. Servizio Idrografico Regionale. Dati Pioggia Gennaio-Dicembre 2022 e 2023. Dati Termometrici (min. med. max.) Gennaio-Dicembre 2022 e 2023. https://www.regione.umbria.it/ambiente/servizio-idrografico.

[29] Atencio S., Verkempinck S.H.E., Bernaerts T., Reineke K., Hendrickx M., Van Loey A. (2022). Impact of processing on the production of a carotenoid-rich Cucurbita maxima cv Hokkaido pumpkin juice. Food Chem..

[30] Regulation (EU) No 1169/2011 of the European Parliament and of the Council of 25 October 2011 on the Provision of Food Information to Consumers. https://eur-lex.europa.eu/eli/reg/2011/1169/oj/eng.

[31] US Department of Agriculture (USDA) Global Agricultural Information Network (GAIN) Report Global Agricultural Information Network. https://www.fas.usda.gov/data.

[32] Kralik G., Kralik Z., Grčević M., Hanžek D., Yücel B., Taşkin T. (2018). Animal husbandry and nutrition. Quality of Chicken Meat.

[33] Santos E.M., Rodriguez J.A., Lorenzo J.M., Mondragón A.C., Pateiro M., Gutiérrez E., Ferreira T.A. (2022). Antioxidant effect of pumpkin flower (*Cucurbita maxima*) in chicken patties. Foods.

[34] Grispoldi L., Ianni F., Blasi F., Pollini L., Crotti S., Cruciani D., Cenci-Goga B.T., Cossignani L. (2022). Apple pomace as valuable food ingredient for enhancing nutritional and antioxidant properties of Italian salami. Antioxidants.

[35] Mangiapelo L., Ianni F., Pagano C., Grispoldi L., Blasi F., Cenci-Goga B.T., Perioli L., Cossignani L. (2023). Role of apple pomace in the formulation of a novel healthy mayonnaise. Eur. Food Res. Technol..

[36] Zargar F.A., Kumar S., Bhat Z.F., Kumar P. (2014). Effect of pumpkin on the quality characteristics and storage quality of aerobically packaged chicken sausages. SpringerPlus.

[37] Vandorou M., Plakidis C., Tsompanidou I.M., Adamantidi T., Panagopoulou E.A., Tsoupras A. (2024). A review on apple pomace bioactives for natural functional food and cosmetic products with therapeutic health-promoting properties. Int. J. Mol. Sci..

[38] Bhushan S., Kalia K., Sharma M., Singh B., Ahuja P.S. (2008). Processing of apple pomace for bioactive molecules. Crit. Rev. Biotechnol..

[39] Koishybayeva A., Korzeniowska M. (2024). Utilization and effect of apple pomace powder on quality characteristics of turkey sausages. Foods.

[40] Rani R., Yadav S., Thakur N., Kumar S., Han H., Alturki H.A., Ahmad M.d.F., Raposo A. (2024). Effect of incorporation of pumpkin seed powder and chia seed powder on storage stability of fiber enriched chicken meat nuggets. LWT-Food Sci. Technol..

