# Supplementary material for: Development of Value-Added Chicken Burgers by Adding Pumpkin Peel Powder as a Sustainable Ingredient
Source: Antioxidants (Basel). 2025 May 28;14(6):648. doi: 10.3390/antiox14060648 (PMC12190050; doi:10.3390/antiox14060648)
Supplement: Supplementary file 1 [file antioxidants-14-00648-s001.zip › ANTIOX_Tables-SupMat.pdf]

SUPPLEMENTARY MATERIAL

Development of value-added chicken burgers by adding pumpkin peel as a sustainable ingredient

Nicola Pinna [1,†], Federica Ianni [1,†], Michela Codini [1], Beniamino Cenci-Goga [2,3], Marco Misuraca [2], Egidia Costanzi [2], Lina Cossignani [1,*] and Francesca Blasi [1]

[1] Department of Pharmaceutical Sciences, University of Perugia, 06126 Perugia, Italy; nicola.pinna@dottorandi.unipg.it (N.P.); federica.ianni@unipg.it (F.I.); michela.codini@unipg.it (M.C.); francesca.blasi@unipg.it (F.B.)

[2] Department of Veterinary Medicine, University of Perugia, 06126 Perugia, Italy; beniamino.cencigoga@unipg.it (B.T.C.-G.); marco.misuraca.tdp@gmail.com (M.M.); egidia.costanzi@unipg.it (E.C.)

[3] Faculty of Veterinary Science, Department of Paraclinical Sciences, University of Pretoria, Onderstepoort 0110, South Africa

* Correspondence: lina.cossignani@unipg.it (L.C.); Tel.: +39-075-585-7959

† These authors contributed equally to this work.

Table S1a: Statistical analysis carried out on L* parameter of PPP (Tukey's multiple comparisons test) of different pumpkin variety (LN, Lunga di Napoli; MDP, Moscata di Provenza; VR, Violina rugosa; HK, Hokkaido; M, Mantovana) and harvesting years (2022-2023)

Tukey's multiple comparisons test	Mean Diff,	95,00% CI of diff,	Below threshold?	Summary	Adjusted P Value	
VR 22 vs. VR 23	-2,007	-2,047 to -1,967	Yes	****	<0,0001	A-B
VR 22 vs. MDP 22	6,413	6,370 to 6,456	Yes	****	<0,0001	A-C
VR 22 vs. MDP 23	5,853	5,813 to 5,893	Yes	****	<0,0001	A-D
VR 22 vs. HK 22	13,77	13,73 to 13,81	Yes	****	<0,0001	A-E
VR 22 vs. HK 23	8,163	8,123 to 8,203	Yes	****	<0,0001	A-F
VR 22 vs. LN 22	15,64	15,60 to 15,69	Yes	****	<0,0001	A-G
VR 22 vs. LN 23	0,3858	0,3457 to 0,4259	Yes	****	<0,0001	A-H
VR 22 vs. M 22	12,91	12,86 to 12,95	Yes	****	<0,0001	A-I
VR 22 vs. M23	10,69	10,65 to 10,73	Yes	****	<0,0001	A-J
VR 23 vs. MDP 22	8,420	8,380 to 8,460	Yes	****	<0,0001	B-C
VR 23 vs. MDP 23	7,860	7,823 to 7,897	Yes	****	<0,0001	B-D
VR 23 vs. HK 22	15,78	15,74 to 15,82	Yes	****	<0,0001	B-E
VR 23 vs. HK 23	10,17	10,13 to 10,21	Yes	****	<0,0001	B-F
VR 23 vs. LN 22	17,65	17,61 to 17,69	Yes	****	<0,0001	B-G
VR 23 vs. LN 23	2,393	2,355 to 2,430	Yes	****	<0,0001	B-H
VR 23 vs. M 22	14,91	14,87 to 14,95	Yes	****	<0,0001	B-I
VR 23 vs. M23	12,70	12,66 to 12,73	Yes	****	<0,0001	B-J
MDP 22 vs. MDP 23	-0,5600	-0,6001 to -0,5199	Yes	****	<0,0001	C-D
MDP 22 vs. HK 22	7,357	7,314 to 7,400	Yes	****	<0,0001	C-E
MDP 22 vs. HK 23	1,750	1,710 to 1,790	Yes	****	<0,0001	C-F
MDP 22 vs. LN 22	9,230	9,187 to 9,273	Yes	****	<0,0001	C-G
MDP 22 vs. LN 23	-6,028	-6,068 to -5,987	Yes	****	<0,0001	C-H
MDP 22 vs. M 22	6,493	6,450 to 6,536	Yes	****	<0,0001	C-I
MDP 22 vs. M23	4,278	4,237 to 4,318	Yes	****	<0,0001	C-J
MDP 23 vs. HK 22	7,917	7,877 to 7,957	Yes	****	<0,0001	D-E
MDP 23 vs. HK 23	2,310	2,273 to 2,347	Yes	****	<0,0001	D-F
MDP 23 vs. LN 22	9,790	9,750 to 9,830	Yes	****	<0,0001	D-G
MDP 23 vs. LN 23	-5,468	-5,505 to -5,430	Yes	****	<0,0001	D-H
MDP 23 vs. M 22	7,053	7,013 to 7,093	Yes	****	<0,0001	D-I
MDP 23 vs. M23	4,838	4,800 to 4,875	Yes	****	<0,0001	D-J
HK 22 vs. HK 23	-5,607	-5,647 to -5,567	Yes	****	<0,0001	E-F
HK 22 vs. LN 22	1,873	1,830 to 1,916	Yes	****	<0,0001	E-G
HK 22 vs. LN 23	-13,38	-13,42 to -13,34	Yes	****	<0,0001	E-H
HK 22 vs. M 22	-0,8633	-0,9062 to -0,8205	Yes	****	<0,0001	E-I
HK 22 vs. M23	-3,079	-3,119 to -3,039	Yes	****	<0,0001	E-J
HK 23 vs. LN 22	7,480	7,440 to 7,520	Yes	****	<0,0001	F-G
HK 23 vs. LN 23	-7,777	-7,815 to -7,740	Yes	****	<0,0001	F-H
HK 23 vs. M 22	4,743	4,703 to 4,783	Yes	****	<0,0001	F-I
HK 23 vs. M23	2,528	2,490 to 2,565	Yes	****	<0,0001	F-J
LN 22 vs. LN 23	-15,26	-15,30 to -15,22	Yes	****	<0,0001	G-H
LN 22 vs. M 22	-2,737	-2,780 to -2,694	Yes	****	<0,0001	G-I
LN 22 vs. M23	-4,953	-4,993 to -4,912	Yes	****	<0,0001	G-J
LN 23 vs. M 22	12,52	12,48 to 12,56	Yes	****	<0,0001	H-I
LN 23 vs. M23	10,31	10,27 to 10,34	Yes	****	<0,0001	H-J
M 22 vs. M23	-2,216	-2,256 to -2,176	Yes	****	<0,0001	I-J

Table S1b: Statistical analysis carried out on a* parameter of PPP (Tukey's multiple comparisons test) of different pumpkin variety (LN, Lunga di Napoli; MDP, Moscata di Provenza; VR, Violina rugosa; HK, Hokkaido; M, Mantovana) and harvesting years (2022-2023)

Tukey's multiple comparisons test	Mean Diff,	95,00% CI of diff,	Below threshold?	Summary	Adjusted P Value	
VR 22 vs. VR 23	0,1425	0,09801 to 0,1870	Yes	****	<0,0001	A-B
VR 22 vs. MDP 22	-1,717	-1,764 to -1,669	Yes	****	<0,0001	A-C
VR 22 vs. MDP 23	-3,940	-3,984 to -3,896	Yes	****	<0,0001	A-D
VR 22 vs. HK 22	-14,14	-14,19 to -14,10	Yes	****	<0,0001	A-E
VR 22 vs. HK 23	-12,29	-12,33 to -12,24	Yes	****	<0,0001	A-F
VR 22 vs. LN 22	0,4967	0,4491 to 0,5442	Yes	****	<0,0001	A-G
VR 22 vs. LN 23	5,650	5,606 to 5,694	Yes	****	<0,0001	A-H
VR 22 vs. M 22	0,4900	0,4424 to 0,5376	Yes	****	<0,0001	A-I
VR 22 vs. M23	7,835	7,791 to 7,879	Yes	****	<0,0001	A-J
VR 23 vs. MDP 22	-1,859	-1,904 to -1,815	Yes	****	<0,0001	B-C
VR 23 vs. MDP 23	-4,083	-4,124 to -4,041	Yes	****	<0,0001	B-D
VR 23 vs. HK 22	-14,29	-14,33 to -14,24	Yes	****	<0,0001	B-E
VR 23 vs. HK 23	-12,43	-12,47 to -12,39	Yes	****	<0,0001	B-F
VR 23 vs. LN 22	0,3542	0,3097 to 0,3987	Yes	****	<0,0001	B-G
VR 23 vs. LN 23	5,508	5,466 to 5,549	Yes	****	<0,0001	B-H
VR 23 vs. M 22	0,3475	0,3030 to 0,3920	Yes	****	<0,0001	B-I
VR 23 vs. M23	7,693	7,651 to 7,734	Yes	****	<0,0001	B-J
MDP 22 vs. MDP 23	-2,223	-2,268 to -2,179	Yes	****	<0,0001	C-D
MDP 22 vs. HK 22	-12,43	-12,47 to -12,38	Yes	****	<0,0001	C-E
MDP 22 vs. HK 23	-10,57	-10,61 to -10,52	Yes	****	<0,0001	C-F
MDP 22 vs. LN 22	2,213	2,166 to 2,261	Yes	****	<0,0001	C-G
MDP 22 vs. LN 23	7,367	7,322 to 7,411	Yes	****	<0,0001	C-H
MDP 22 vs. M 22	2,207	2,159 to 2,254	Yes	****	<0,0001	C-I
MDP 22 vs. M23	9,552	9,507 to 9,596	Yes	****	<0,0001	C-J
MDP 23 vs. HK 22	-10,20	-10,25 to -10,16	Yes	****	<0,0001	D-E
MDP 23 vs. HK 23	-8,345	-8,386 to -8,304	Yes	****	<0,0001	D-F
MDP 23 vs. LN 22	4,437	4,392 to 4,481	Yes	****	<0,0001	D-G
MDP 23 vs. LN 23	9,590	9,549 to 9,631	Yes	****	<0,0001	D-H
MDP 23 vs. M 22	4,430	4,386 to 4,474	Yes	****	<0,0001	D-I
MDP 23 vs. M23	11,78	11,73 to 11,82	Yes	****	<0,0001	D-J
HK 22 vs. HK 23	1,858	1,814 to 1,903	Yes	****	<0,0001	E-F
HK 22 vs. LN 22	14,64	14,59 to 14,69	Yes	****	<0,0001	E-G
HK 22 vs. LN 23	19,79	19,75 to 19,84	Yes	****	<0,0001	E-H
HK 22 vs. M 22	14,63	14,59 to 14,68	Yes	****	<0,0001	E-I
HK 22 vs. M23	21,98	21,93 to 22,02	Yes	****	<0,0001	E-J
HK 23 vs. LN 22	12,78	12,74 to 12,83	Yes	****	<0,0001	F-G
HK 23 vs. LN 23	17,94	17,89 to 17,98	Yes	****	<0,0001	F-H
HK 23 vs. M 22	12,78	12,73 to 12,82	Yes	****	<0,0001	F-I
HK 23 vs. M23	20,12	20,08 to 20,16	Yes	****	<0,0001	F-J
LN 22 vs. LN 23	5,153	5,109 to 5,198	Yes	****	<0,0001	G-H
LN 22 vs. M 22	-0,006667	-0,05423 to 0,04089	No	ns	>0,9999	G-I
LN 22 vs. M23	7,338	7,294 to 7,383	Yes	****	<0,0001	G-J
LN 23 vs. M 22	-5,160	-5,204 to -5,116	Yes	****	<0,0001	H-I
LN 23 vs. M23	2,185	2,144 to 2,226	Yes	****	<0,0001	H-J
M 22 vs. M23	7,345	7,301 to 7,389	Yes	****	<0,0001	I-J

Table S1c: Statistical analysis carried out on b* parameter of PPP (Tukey's multiple comparisons test) of different pumpkin variety (LN, Lunga di Napoli; MDP, Moscata di Provenza; VR, Violina rugosa; HK, Hokkaido; M, Mantovana) and harvesting years (2022-2023)

Tukey's multiple comparisons test	Mean Diff,	95,00% CI of diff,	Below threshold?	Summary	Adjusted P Value	
VR 22 vs. VR 23	-5,207	-5,284 to -5,130	Yes	****	<0,0001	A-B
VR 22 vs. MDP 22	2,520	2,438 to 2,602	Yes	****	<0,0001	A-C
VR 22 vs. MDP 23	5,598	5,521 to 5,675	Yes	****	<0,0001	A-D
VR 22 vs. HK 22	-23,01	-23,10 to -22,93	Yes	****	<0,0001	A-E
VR 22 vs. HK 23	-35,19	-35,27 to -35,11	Yes	****	<0,0001	A-F
VR 22 vs. LN 22	-1,853	-1,935 to -1,771	Yes	****	<0,0001	A-G
VR 22 vs. LN 23	-3,992	-4,069 to -3,915	Yes	****	<0,0001	A-H
VR 22 vs. M 22	-3,033	-3,115 to -2,951	Yes	****	<0,0001	A-I
VR 22 vs. M23	-21,54	-21,62 to -21,47	Yes	****	<0,0001	A-J
VR 23 vs. MDP 22	7,727	7,650 to 7,804	Yes	****	<0,0001	B-C
VR 23 vs. MDP 23	10,81	10,73 to 10,88	Yes	****	<0,0001	B-D
VR 23 vs. HK 22	-17,81	-17,88 to -17,73	Yes	****	<0,0001	B-E
VR 23 vs. HK 23	-29,99	-30,06 to -29,91	Yes	****	<0,0001	B-F
VR 23 vs. LN 22	3,353	3,276 to 3,430	Yes	****	<0,0001	B-G
VR 23 vs. LN 23	1,215	1,144 to 1,286	Yes	****	<0,0001	B-H
VR 23 vs. M 22	2,173	2,096 to 2,250	Yes	****	<0,0001	B-I
VR 23 vs. M23	-16,34	-16,41 to -16,27	Yes	****	<0,0001	B-J
MDP 22 vs. MDP 23	3,078	3,001 to 3,155	Yes	****	<0,0001	C-D
MDP 22 vs. HK 22	-25,53	-25,62 to -25,45	Yes	****	<0,0001	C-E
MDP 22 vs. HK 23	-37,71	-37,79 to -37,63	Yes	****	<0,0001	C-F
MDP 22 vs. LN 22	-4,373	-4,455 to -4,291	Yes	****	<0,0001	C-G
MDP 22 vs. LN 23	-6,512	-6,589 to -6,435	Yes	****	<0,0001	C-H
MDP 22 vs. M 22	-5,553	-5,635 to -5,471	Yes	****	<0,0001	C-I
MDP 22 vs. M23	-24,06	-24,14 to -23,99	Yes	****	<0,0001	C-J
MDP 23 vs. HK 22	-28,61	-28,69 to -28,53	Yes	****	<0,0001	D-E
MDP 23 vs. HK 23	-40,79	-40,86 to -40,72	Yes	****	<0,0001	D-F
MDP 23 vs. LN 22	-7,452	-7,529 to -7,375	Yes	****	<0,0001	D-G
MDP 23 vs. LN 23	-9,590	-9,661 to -9,519	Yes	****	<0,0001	D-H
MDP 23 vs. M 22	-8,632	-8,709 to -8,555	Yes	****	<0,0001	D-I
MDP 23 vs. M23	-27,14	-27,21 to -27,07	Yes	****	<0,0001	D-J
HK 22 vs. HK 23	-12,18	-12,26 to -12,10	Yes	****	<0,0001	E-F
HK 22 vs. LN 22	21,16	21,08 to 21,24	Yes	****	<0,0001	E-G
HK 22 vs. LN 23	19,02	18,94 to 19,10	Yes	****	<0,0001	E-H
HK 22 vs. M 22	19,98	19,90 to 20,06	Yes	****	<0,0001	E-I
HK 22 vs. M23	1,469	1,392 to 1,546	Yes	****	<0,0001	E-J
HK 23 vs. LN 22	33,34	33,26 to 33,42	Yes	****	<0,0001	F-G
HK 23 vs. LN 23	31,20	31,13 to 31,27	Yes	****	<0,0001	F-H
HK 23 vs. M 22	32,16	32,08 to 32,24	Yes	****	<0,0001	F-I
HK 23 vs. M23	13,65	13,58 to 13,72	Yes	****	<0,0001	F-J
LN 22 vs. LN 23	-2,138	-2,215 to -2,061	Yes	****	<0,0001	G-H
LN 22 vs. M 22	-1,180	-1,262 to -1,098	Yes	****	<0,0001	G-I
LN 22 vs. M23	-19,69	-19,77 to -19,61	Yes	****	<0,0001	G-J
LN 23 vs. M 22	0,9583	0,8815 to 1,035	Yes	****	<0,0001	H-I
LN 23 vs. M23	-17,55	-17,62 to -17,48	Yes	****	<0,0001	H-J
M 22 vs. M23	-18,51	-18,59 to -18,43	Yes	****	<0,0001	I-J

Table S2a: Statistical analysis carried out on TCC of PPP (Tukey's multiple comparisons test) of different pumpkin variety (LN, Lunga di Napoli; MDP, Moscata di Provenza; VR, Violina rugosa; HK, Hokkaido; M, Mantovana) and harvesting years (2022-2023)

Tukey's multiple comparisons test	Mean Diff,	95,00% CI of diff,	Below threshold?	Summary	Adjusted P Value	
VR 22 vs. VR 23	86,75	-61,97 to 235,5	No	ns	0,4541	A-B
VR 22 vs. MDP 22	159,6	10,90 to 308,3	Yes	*	0,0330	A-C
VR 22 vs. MDP 23	170,7	21,95 to 319,4	Yes	*	0,0217	A-D
VR 22 vs. HK 22	-2692	-2841 to -2544	Yes	****	<0,0001	A-E
VR 22 vs. HK 23	-1630	-1779 to -1481	Yes	****	<0,0001	A-F
VR 22 vs. LN 22	-358,2	-506,9 to -209,5	Yes	****	<0,0001	A-G
VR 22 vs. LN 23	-600,3	-749,0 to -451,5	Yes	****	<0,0001	A-H
VR 22 vs. M 22	-626,5	-775,2 to -477,8	Yes	****	<0,0001	A-I
VR 22 vs. M23	-1122	-1271 to -973,2	Yes	****	<0,0001	A-J
VR 23 vs. MDP 22	72,88	-75,84 to 221,6	No	ns	0,6493	B-C
VR 23 vs. MDP 23	83,92	-64,80 to 232,6	No	ns	0,4919	B-D
VR 23 vs. HK 22	-2779	-2928 to -2630	Yes	****	<0,0001	B-E
VR 23 vs. HK 23	-1717	-1865 to -1568	Yes	****	<0,0001	B-F
VR 23 vs. LN 22	-444,9	-593,6 to -296,2	Yes	****	<0,0001	B-G
VR 23 vs. LN 23	-687,0	-835,7 to -538,3	Yes	****	<0,0001	B-H
VR 23 vs. M 22	-713,3	-862,0 to -564,6	Yes	****	<0,0001	B-I
VR 23 vs. M23	-1209	-1357 to -1060	Yes	****	<0,0001	B-J
MDP 22 vs. MDP 23	11,05	-137,7 to 159,8	No	ns	>0,9999	C-D
MDP 22 vs. HK 22	-2852	-3001 to -2703	Yes	****	<0,0001	C-E
MDP 22 vs. HK 23	-1790	-1938 to -1641	Yes	****	<0,0001	C-F
MDP 22 vs. LN 22	-517,8	-666,5 to -369,1	Yes	****	<0,0001	C-G
MDP 22 vs. LN 23	-759,9	-908,6 to -611,2	Yes	****	<0,0001	C-H
MDP 22 vs. M 22	-786,2	-934,9 to -637,4	Yes	****	<0,0001	C-I
MDP 22 vs. M23	-1282	-1430 to -1133	Yes	****	<0,0001	C-J
MDP 23 vs. HK 22	-2863	-3012 to -2714	Yes	****	<0,0001	D-E
MDP 23 vs. HK 23	-1801	-1949 to -1652	Yes	****	<0,0001	D-F
MDP 23 vs. LN 22	-528,8	-677,6 to -380,1	Yes	****	<0,0001	D-G
MDP 23 vs. LN 23	-770,9	-919,6 to -622,2	Yes	****	<0,0001	D-H
MDP 23 vs. M 22	-797,2	-945,9 to -648,5	Yes	****	<0,0001	D-I
MDP 23 vs. M23	-1293	-1441 to -1144	Yes	****	<0,0001	D-J
HK 22 vs. HK 23	1063	913,8 to 1211	Yes	****	<0,0001	E-F
HK 22 vs. LN 22	2334	2186 to 2483	Yes	****	<0,0001	E-G
HK 22 vs. LN 23	2092	1943 to 2241	Yes	****	<0,0001	E-H
HK 22 vs. M 22	2066	1917 to 2215	Yes	****	<0,0001	E-I
HK 22 vs. M23	1571	1422 to 1719	Yes	****	<0,0001	E-J
HK 23 vs. LN 22	1272	1123 to 1420	Yes	****	<0,0001	F-G
HK 23 vs. LN 23	1030	880,9 to 1178	Yes	****	<0,0001	F-H
HK 23 vs. M 22	1003	854,7 to 1152	Yes	****	<0,0001	F-I
HK 23 vs. M23	508,0	359,3 to 656,7	Yes	****	<0,0001	F-J
LN 22 vs. LN 23	-242,1	-390,8 to -93,37	Yes	**	0,0017	G-H
LN 22 vs. M 22	-268,4	-417,1 to -119,6	Yes	***	0,0008	G-I
LN 22 vs. M23	-763,7	-912,5 to -615,0	Yes	****	<0,0001	G-J
LN 23 vs. M 22	-26,28	-175,0 to 122,4	No	ns	0,9988	H-I
LN 23 vs. M23	-521,7	-670,4 to -372,9	Yes	****	<0,0001	H-J
M 22 vs. M23	-495,4	-644,1 to -346,7	Yes	****	<0,0001	I-J

Table S2b: Statistical analysis carried out on ABTS of PPP (Tukey's multiple comparisons test) of different pumpkin variety (LN, Lunga di Napoli; MDP, Moscata di Provenza; VR, Violina rugosa; HK, Hokkaido; M, Mantovana) and harvesting years (2022-2023)

Tukey's multiple comparisons test	Mean Diff,	95,00% CI of diff,	Below threshold?	Summary	Adjusted P Value	
VR 22 vs. VR 23	-114,7	-162,9 to -66,57	Yes	****	<0,0001	A-B
VR 22 vs. MDP 22	-221,6	-269,8 to -173,5	Yes	****	<0,0001	A-C
VR 22 vs. MDP 23	-651,7	-699,9 to -603,6	Yes	****	<0,0001	A-D
VR 22 vs. HK 22	-1860	-1909 to -1812	Yes	****	<0,0001	A-E
VR 22 vs. HK 23	-1136	-1184 to -1088	Yes	****	<0,0001	A-F
VR 22 vs. LN 22	-3029	-3077 to -2981	Yes	****	<0,0001	A-G
VR 22 vs. LN 23	-2413	-2461 to -2365	Yes	****	<0,0001	A-H
VR 22 vs. M 22	-815,6	-863,8 to -767,4	Yes	****	<0,0001	A-I
VR 22 vs. M23	-1473	-1521 to -1425	Yes	****	<0,0001	A-J
VR 23 vs. MDP 22	-106,9	-155,1 to -58,73	Yes	***	0,0001	B-C
VR 23 vs. MDP 23	-537,0	-585,2 to -488,8	Yes	****	<0,0001	B-D
VR 23 vs. HK 22	-1746	-1794 to -1698	Yes	****	<0,0001	B-E
VR 23 vs. HK 23	-1021	-1069 to -973,0	Yes	****	<0,0001	B-F
VR 23 vs. LN 22	-2914	-2962 to -2866	Yes	****	<0,0001	B-G
VR 23 vs. LN 23	-2298	-2346 to -2250	Yes	****	<0,0001	B-H
VR 23 vs. M 22	-700,9	-749,0 to -652,7	Yes	****	<0,0001	B-I
VR 23 vs. M23	-1358	-1406 to -1310	Yes	****	<0,0001	B-J
MDP 22 vs. MDP 23	-430,1	-478,3 to -381,9	Yes	****	<0,0001	C-D
MDP 22 vs. HK 22	-1639	-1687 to -1591	Yes	****	<0,0001	C-E
MDP 22 vs. HK 23	-914,3	-962,4 to -866,1	Yes	****	<0,0001	C-F
MDP 22 vs. LN 22	-2807	-2855 to -2759	Yes	****	<0,0001	C-G
MDP 22 vs. LN 23	-2191	-2240 to -2143	Yes	****	<0,0001	C-H
MDP 22 vs. M 22	-594,0	-642,1 to -545,8	Yes	****	<0,0001	C-I
MDP 22 vs. M23	-1251	-1299 to -1203	Yes	****	<0,0001	C-J
MDP 23 vs. HK 22	-1209	-1257 to -1161	Yes	****	<0,0001	D-E
MDP 23 vs. HK 23	-484,1	-532,3 to -436,0	Yes	****	<0,0001	D-F
MDP 23 vs. LN 22	-2377	-2425 to -2329	Yes	****	<0,0001	D-G
MDP 23 vs. LN 23	-1761	-1809 to -1713	Yes	****	<0,0001	D-H
MDP 23 vs. M 22	-163,8	-212,0 to -115,7	Yes	****	<0,0001	D-I
MDP 23 vs. M23	-821,0	-869,2 to -772,8	Yes	****	<0,0001	D-J
HK 22 vs. HK 23	724,6	676,4 to 772,7	Yes	****	<0,0001	E-F
HK 22 vs. LN 22	-1168	-1217 to -1120	Yes	****	<0,0001	E-G
HK 22 vs. LN 23	-552,6	-600,8 to -504,4	Yes	****	<0,0001	E-H
HK 22 vs. M 22	1045	996,7 to 1093	Yes	****	<0,0001	E-I
HK 22 vs. M23	387,7	339,5 to 435,9	Yes	****	<0,0001	E-J
HK 23 vs. LN 22	-1893	-1941 to -1845	Yes	****	<0,0001	F-G
HK 23 vs. LN 23	-1277	-1325 to -1229	Yes	****	<0,0001	F-H
HK 23 vs. M 22	320,3	272,1 to 368,5	Yes	****	<0,0001	F-I
HK 23 vs. M23	-336,9	-385,0 to -288,7	Yes	****	<0,0001	F-J
LN 22 vs. LN 23	615,8	567,6 to 664,0	Yes	****	<0,0001	G-H
LN 22 vs. M 22	2213	2165 to 2261	Yes	****	<0,0001	G-I
LN 22 vs. M23	1556	1508 to 1604	Yes	****	<0,0001	G-J
LN 23 vs. M 22	1597	1549 to 1646	Yes	****	<0,0001	H-I
LN 23 vs. M23	940,3	892,1 to 988,5	Yes	****	<0,0001	H-J
M 22 vs. M23	-657,1	-705,3 to -609,0	Yes	****	<0,0001	I-J

Table S2c: Statistical analysis carried out on ORAC of PPP (Tukey's multiple comparisons test) of different pumpkin variety (LN, Lunga di Napoli; MDP, Moscata di Provenza; VR, Violina rugosa; HK, Hokkaido; M, Mantovana) and harvesting years (2022-2023)

Tukey's multiple comparisons test	Mean Diff,	95,00% CI of diff,	Below threshold?	Summary	Adjusted P Value	
VR 22 vs. VR 23	-426,6	-605,5 to -247,6	Yes	****	<0,0001	A-B
VR 22 vs. MDP 22	-824,0	-1003 to -645,1	Yes	****	<0,0001	A-C
VR 22 vs. MDP 23	-2423	-2602 to -2244	Yes	****	<0,0001	A-D
VR 22 vs. HK 22	-6917	-7096 to -6738	Yes	****	<0,0001	A-E
VR 22 vs. HK 23	-4223	-4402 to -4044	Yes	****	<0,0001	A-F
VR 22 vs. LN 22	-11261	-11440 to -11082	Yes	****	<0,0001	A-G
VR 22 vs. LN 23	-8972	-9151 to -8793	Yes	****	<0,0001	A-H
VR 22 vs. M 22	-3032	-3211 to -2853	Yes	****	<0,0001	A-I
VR 22 vs. M23	-5476	-5655 to -5297	Yes	****	<0,0001	A-J
VR 23 vs. MDP 22	-397,4	-576,4 to -218,5	Yes	***	0,0001	B-C
VR 23 vs. MDP 23	-1997	-2176 to -1818	Yes	****	<0,0001	B-D
VR 23 vs. HK 22	-6490	-6669 to -6311	Yes	****	<0,0001	B-E
VR 23 vs. HK 23	-3797	-3975 to -3618	Yes	****	<0,0001	B-F
VR 23 vs. LN 22	-10835	-11014 to -10656	Yes	****	<0,0001	B-G
VR 23 vs. LN 23	-8545	-8724 to -8366	Yes	****	<0,0001	B-H
VR 23 vs. M 22	-2606	-2785 to -2427	Yes	****	<0,0001	B-I
VR 23 vs. M23	-5049	-5228 to -4870	Yes	****	<0,0001	B-J
MDP 22 vs. MDP 23	-1599	-1778 to -1420	Yes	****	<0,0001	C-D
MDP 22 vs. HK 22	-6093	-6272 to -5914	Yes	****	<0,0001	C-E
MDP 22 vs. HK 23	-3399	-3578 to -3220	Yes	****	<0,0001	C-F
MDP 22 vs. LN 22	-10437	-10616 to -10258	Yes	****	<0,0001	C-G
MDP 22 vs. LN 23	-8148	-8327 to -7969	Yes	****	<0,0001	C-H
MDP 22 vs. M 22	-2208	-2387 to -2029	Yes	****	<0,0001	C-I
MDP 22 vs. M23	-4652	-4831 to -4473	Yes	****	<0,0001	C-J
MDP 23 vs. HK 22	-4494	-4673 to -4315	Yes	****	<0,0001	D-E
MDP 23 vs. HK 23	-1800	-1979 to -1621	Yes	****	<0,0001	D-F
MDP 23 vs. LN 22	-8838	-9017 to -8659	Yes	****	<0,0001	D-G
MDP 23 vs. LN 23	-6549	-6727 to -6370	Yes	****	<0,0001	D-H
MDP 23 vs. M 22	-609,2	-788,1 to -430,2	Yes	****	<0,0001	D-I
MDP 23 vs. M23	-3053	-3232 to -2874	Yes	****	<0,0001	D-J
HK 22 vs. HK 23	2694	2515 to 2873	Yes	****	<0,0001	E-F
HK 22 vs. LN 22	-4344	-4523 to -4165	Yes	****	<0,0001	E-G
HK 22 vs. LN 23	-2055	-2234 to -1876	Yes	****	<0,0001	E-H
HK 22 vs. M 22	3885	3706 to 4064	Yes	****	<0,0001	E-I
HK 22 vs. M23	1441	1262 to 1620	Yes	****	<0,0001	E-J
HK 23 vs. LN 22	-7038	-7217 to -6859	Yes	****	<0,0001	F-G
HK 23 vs. LN 23	-4749	-4928 to -4570	Yes	****	<0,0001	F-H
HK 23 vs. M 22	1191	1012 to 1370	Yes	****	<0,0001	F-I
HK 23 vs. M23	-1253	-1432 to -1074	Yes	****	<0,0001	F-J
LN 22 vs. LN 23	2290	2111 to 2469	Yes	****	<0,0001	G-H
LN 22 vs. M 22	8229	8050 to 8408	Yes	****	<0,0001	G-I
LN 22 vs. M23	5786	5607 to 5964	Yes	****	<0,0001	G-J
LN 23 vs. M 22	5939	5760 to 6118	Yes	****	<0,0001	H-I
LN 23 vs. M23	3496	3317 to 3675	Yes	****	<0,0001	H-J
M 22 vs. M23	-2443	-2622 to -2264	Yes	****	<0,0001	I-J

Table S3a: Statistical analysis carried out on HPLC data of free xanthophylls (Tukey's multiple comparisons test) of different pumpkin variety (LN, Lunga di Napoli; MDP, Moscata di Provenza; VR, Violina rugosa; HK, Hokkaido; M, Mantovana) and harvesting years (2022-2023)

Tukey's multiple comparisons test	Mean Diff,	95,00% CI of diff,	Below threshold?	Summary	Adjusted P Value	
VR 22 vs. VR 23	-0,3500	-28,60 to 27,90	No	ns	>0,9999	A-B
VR 22 vs. MDP 22	1,760	-26,49 to 30,01	No	ns	>0,9999	A-C
VR 22 vs. MDP 23	0,9750	-27,27 to 29,22	No	ns	>0,9999	A-D
VR 22 vs. HK 22	-618,4	-646,7 to -590,2	Yes	****	<0,0001	A-E
VR 22 vs. HK 23	-364,5	-392,7 to -336,2	Yes	****	<0,0001	A-F
VR 22 vs. LN 22	-97,27	-125,5 to -69,02	Yes	****	<0,0001	A-G
VR 22 vs. LN 23	-90,00	-118,2 to -61,75	Yes	****	<0,0001	A-H
VR 22 vs. M 22	-219,9	-248,1 to -191,6	Yes	****	<0,0001	A-I
VR 22 vs. M23	-198,8	-227,1 to -170,6	Yes	****	<0,0001	A-J
VR 23 vs. MDP 22	2,110	-26,14 to 30,36	No	ns	>0,9999	B-C
VR 23 vs. MDP 23	1,325	-26,92 to 29,57	No	ns	>0,9999	B-D
VR 23 vs. HK 22	-618,1	-646,3 to -589,8	Yes	****	<0,0001	B-E
VR 23 vs. HK 23	-364,1	-392,3 to -335,9	Yes	****	<0,0001	B-F
VR 23 vs. LN 22	-96,92	-125,2 to -68,67	Yes	****	<0,0001	B-G
VR 23 vs. LN 23	-89,65	-117,9 to -61,40	Yes	****	<0,0001	B-H
VR 23 vs. M 22	-219,5	-247,8 to -191,3	Yes	****	<0,0001	B-I
VR 23 vs. M23	-198,5	-226,7 to -170,2	Yes	****	<0,0001	B-J
MDP 22 vs. MDP 23	-0,7850	-29,03 to 27,46	No	ns	>0,9999	C-D
MDP 22 vs. HK 22	-620,2	-648,4 to -591,9	Yes	****	<0,0001	C-E
MDP 22 vs. HK 23	-366,2	-394,5 to -338,0	Yes	****	<0,0001	C-F
MDP 22 vs. LN 22	-99,03	-127,3 to -70,78	Yes	****	<0,0001	C-G
MDP 22 vs. LN 23	-91,76	-120,0 to -63,51	Yes	****	<0,0001	C-H
MDP 22 vs. M 22	-221,7	-249,9 to -193,4	Yes	****	<0,0001	C-I
MDP 22 vs. M23	-200,6	-228,8 to -172,3	Yes	****	<0,0001	C-J
MDP 23 vs. HK 22	-619,4	-647,6 to -591,1	Yes	****	<0,0001	D-E
MDP 23 vs. HK 23	-365,4	-393,7 to -337,2	Yes	****	<0,0001	D-F
MDP 23 vs. LN 22	-98,25	-126,5 to -70,00	Yes	****	<0,0001	D-G
MDP 23 vs. LN 23	-90,98	-119,2 to -62,73	Yes	****	<0,0001	D-H
MDP 23 vs. M 22	-220,9	-249,1 to -192,6	Yes	****	<0,0001	D-I
MDP 23 vs. M23	-199,8	-228,1 to -171,6	Yes	****	<0,0001	D-J
HK 22 vs. HK 23	254,0	225,7 to 282,2	Yes	****	<0,0001	E-F
HK 22 vs. LN 22	521,2	492,9 to 549,4	Yes	****	<0,0001	E-G
HK 22 vs. LN 23	528,4	500,2 to 556,7	Yes	****	<0,0001	E-H
HK 22 vs. M 22	398,5	370,3 to 426,8	Yes	****	<0,0001	E-I
HK 22 vs. M23	419,6	391,3 to 447,8	Yes	****	<0,0001	E-J
HK 23 vs. LN 22	267,2	238,9 to 295,4	Yes	****	<0,0001	F-G
HK 23 vs. LN 23	274,5	246,2 to 302,7	Yes	****	<0,0001	F-H
HK 23 vs. M 22	144,6	116,3 to 172,8	Yes	****	<0,0001	F-I
HK 23 vs. M23	165,6	137,4 to 193,9	Yes	****	<0,0001	F-J
LN 22 vs. LN 23	7,270	-20,98 to 35,52	No	ns	0,9834	G-H
LN 22 vs. M 22	-122,6	-150,9 to -94,38	Yes	****	<0,0001	G-I
LN 22 vs. M23	-101,6	-129,8 to -73,31	Yes	****	<0,0001	G-J
LN 23 vs. M 22	-129,9	-158,1 to -101,6	Yes	****	<0,0001	H-I
LN 23 vs. M23	-108,8	-137,1 to -80,58	Yes	****	<0,0001	H-J
M 22 vs. M23	21,07	-7,180 to 49,31	No	ns	0,2060	I-J

Table S3b: Statistical analysis carried out on HPLC data of monoesterified xanthophylls (Tukey's multiple comparisons test) of different pumpkin variety (LN, Lunga di Napoli; MDP, Moscata di Provenza; VR, Violina rugosa; HK, Hokkaido; M, Mantovana) and harvesting years (2022-2023)

Tukey's multiple comparisons test	Mean Diff,	95,00% CI of diff,	Below threshold?	Summary	Adjusted P Value
VR 22 vs. VR 23	63,62	-546,8 to 674,1	No	ns	>0,9999
VR 22 vs. MDP 22	107,2	-503,3 to 717,6	No	ns	0,9989
VR 22 vs. MDP 23	123,4	-487,1 to 733,8	No	ns	0,9968
VR 22 vs. HK 22	-18640	-19250 to -18030	Yes	****	<0,0001
VR 22 vs. HK 23	-8835	-9445 to -8224	Yes	****	<0,0001
VR 22 vs. LN 22	-694,8	-1305 to -84,31	Yes	*	0,0229
VR 22 vs. LN 23	-421,7	-1032 to 188,8	No	ns	0,2739
VR 22 vs. M 22	-1802	-2413 to -1192	Yes	****	<0,0001
VR 22 vs. M23	-3738	-4348 to -3127	Yes	****	<0,0001
VR 23 vs. MDP 22	43,56	-566,9 to 654,0	No	ns	>0,9999
VR 23 vs. MDP 23	59,76	-550,7 to 670,2	No	ns	>0,9999
VR 23 vs. HK 22	-18704	-19314 to -18093	Yes	****	<0,0001
VR 23 vs. HK 23	-8898	-9509 to -8288	Yes	****	<0,0001
VR 23 vs. LN 22	-758,4	-1369 to -147,9	Yes	*	0,0129
VR 23 vs. LN 23	-485,3	-1096 to 125,2	No	ns	0,1581
VR 23 vs. M 22	-1866	-2476 to -1255	Yes	****	<0,0001
VR 23 vs. M23	-3801	-4412 to -3191	Yes	****	<0,0001
MDP 22 vs. MDP 23	16,20	-594,3 to 626,7	No	ns	>0,9999
MDP 22 vs. HK 22	-18747	-19358 to -18137	Yes	****	<0,0001
MDP 22 vs. HK 23	-8942	-9553 to -8332	Yes	****	<0,0001
MDP 22 vs. LN 22	-802,0	-1412 to -191,5	Yes	**	0,0087
MDP 22 vs. LN 23	-528,9	-1139 to 81,60	No	ns	0,1065
MDP 22 vs. M 22	-1909	-2520 to -1299	Yes	****	<0,0001
MDP 22 vs. M23	-3845	-4455 to -3235	Yes	****	<0,0001
MDP 23 vs. HK 22	-18763	-19374 to -18153	Yes	****	<0,0001
MDP 23 vs. HK 23	-8958	-9569 to -8348	Yes	****	<0,0001
MDP 23 vs. LN 22	-818,2	-1429 to -207,7	Yes	**	0,0076
MDP 23 vs. LN 23	-545,1	-1156 to 65,40	No	ns	0,0918
MDP 23 vs. M 22	-1926	-2536 to -1315	Yes	****	<0,0001
MDP 23 vs. M23	-3861	-4472 to -3251	Yes	****	<0,0001
HK 22 vs. HK 23	9805	9195 to 10416	Yes	****	<0,0001
HK 22 vs. LN 22	17945	17335 to 18556	Yes	****	<0,0001
HK 22 vs. LN 23	18218	17608 to 18829	Yes	****	<0,0001
HK 22 vs. M 22	16838	16227 to 17448	Yes	****	<0,0001
HK 22 vs. M23	14902	14292 to 15513	Yes	****	<0,0001
HK 23 vs. LN 22	8140	7530 to 8751	Yes	****	<0,0001
HK 23 vs. LN 23	8413	7803 to 9024	Yes	****	<0,0001
HK 23 vs. M 22	7033	6422 to 7643	Yes	****	<0,0001
HK 23 vs. M23	5097	4487 to 5708	Yes	****	<0,0001
LN 22 vs. LN 23	273,1	-337,4 to 883,6	No	ns	0,7394
LN 22 vs. M 22	-1107	-1718 to -497,0	Yes	***	0,0007
LN 22 vs. M23	-3043	-3653 to -2433	Yes	****	<0,0001
LN 23 vs. M 22	-1381	-1991 to -770,1	Yes	***	0,0001
LN 23 vs. M23	-3316	-3927 to -2706	Yes	****	<0,0001
M 22 vs. M23	-1936	-2546 to -1325	Yes	****	<0,0001

Table S3c: Statistical analysis carried out on HPLC data of diesterified xanthophylls (Tukey's multiple comparisons test) of different pumpkin variety (LN, Lunga di Napoli; MDP, Moscata di Provenza; VR, Violina rugosa; HK, Hokkaido; M, Mantovana) and harvesting years (2022-2023)

Tukey's multiple comparisons test	Mean Diff,	95,00% CI of diff,	Below threshold?	Summary	Adjusted P Value
VR 22 vs. VR 23	5,810	-118,5 to 130,1	No	ns	>0,9999
VR 22 vs. MDP 22	265,9	141,6 to 390,2	Yes	***	0,0002
VR 22 vs. MDP 23	243,1	118,8 to 367,3	Yes	***	0,0004
VR 22 vs. HK 22	-1998	-2122 to -1873	Yes	****	<0,0001
VR 22 vs. HK 23	-1533	-1657 to -1408	Yes	****	<0,0001
VR 22 vs. LN 22	-686,9	-811,1 to -562,6	Yes	****	<0,0001
VR 22 vs. LN 23	-637,1	-761,4 to -512,9	Yes	****	<0,0001
VR 22 vs. M 22	-95,47	-219,7 to 28,80	No	ns	0,1828
VR 22 vs. M23	-779,4	-903,7 to -655,1	Yes	****	<0,0001
VR 23 vs. MDP 22	260,1	135,8 to 384,4	Yes	***	0,0002
VR 23 vs. MDP 23	237,2	113,0 to 361,5	Yes	***	0,0005
VR 23 vs. HK 22	-2003	-2128 to -1879	Yes	****	<0,0001
VR 23 vs. HK 23	-1539	-1663 to -1414	Yes	****	<0,0001
VR 23 vs. LN 22	-692,7	-817,0 to -568,4	Yes	****	<0,0001
VR 23 vs. LN 23	-643,0	-767,2 to -518,7	Yes	****	<0,0001
VR 23 vs. M 22	-101,3	-225,5 to 22,99	No	ns	0,1417
VR 23 vs. M23	-785,2	-909,5 to -660,9	Yes	****	<0,0001
MDP 22 vs. MDP 23	-22,86	-147,1 to 101,4	No	ns	0,9984
MDP 22 vs. HK 22	-2264	-2388 to -2139	Yes	****	<0,0001
MDP 22 vs. HK 23	-1799	-1923 to -1674	Yes	****	<0,0001
MDP 22 vs. LN 22	-952,8	-1077 to -828,5	Yes	****	<0,0001
MDP 22 vs. LN 23	-903,1	-1027 to -778,8	Yes	****	<0,0001
MDP 22 vs. M 22	-361,4	-485,6 to -237,1	Yes	****	<0,0001
MDP 22 vs. M23	-1045	-1170 to -921,0	Yes	****	<0,0001
MDP 23 vs. HK 22	-2241	-2365 to -2116	Yes	****	<0,0001
MDP 23 vs. HK 23	-1776	-1900 to -1652	Yes	****	<0,0001
MDP 23 vs. LN 22	-929,9	-1054 to -805,7	Yes	****	<0,0001
MDP 23 vs. LN 23	-880,2	-1004 to -755,9	Yes	****	<0,0001
MDP 23 vs. M 22	-338,5	-462,8 to -214,3	Yes	****	<0,0001
MDP 23 vs. M23	-1022	-1147 to -898,2	Yes	****	<0,0001
HK 22 vs. HK 23	464,9	340,6 to 589,1	Yes	****	<0,0001
HK 22 vs. LN 22	1311	1186 to 1435	Yes	****	<0,0001
HK 22 vs. LN 23	1360	1236 to 1485	Yes	****	<0,0001
HK 22 vs. M 22	1902	1778 to 2026	Yes	****	<0,0001
HK 22 vs. M23	1218	1094 to 1343	Yes	****	<0,0001
HK 23 vs. LN 22	845,9	721,6 to 970,2	Yes	****	<0,0001
HK 23 vs. LN 23	895,6	771,4 to 1020	Yes	****	<0,0001
HK 23 vs. M 22	1437	1313 to 1562	Yes	****	<0,0001
HK 23 vs. M23	753,4	629,1 to 877,6	Yes	****	<0,0001
LN 22 vs. LN 23	49,74	-74,53 to 174,0	No	ns	0,8299
LN 22 vs. M 22	591,4	467,1 to 715,7	Yes	****	<0,0001
LN 22 vs. M23	-92,51	-216,8 to 31,76	No	ns	0,2075
LN 23 vs. M 22	541,7	417,4 to 665,9	Yes	****	<0,0001
LN 23 vs. M23	-142,2	-266,5 to -17,98	Yes	*	0,0221
M 22 vs. M23	-683,9	-808,2 to -559,7	Yes	****	<0,0001

Table S3d: Statistical analysis carried out on HPLC data of β-Carotene (Tukey's multiple comparisons test) of different pumpkin variety (LN, Lunga di Napoli; MDP, Moscata di Provenza; VR, Violina rugosa; HK, Hokkaido; M, Mantovana) and harvesting years (2022-2023)

Tukey's multiple comparisons test	Mean Diff,	95,00% CI of diff,	Below threshold?	Summary	Adjusted P Value
VR 22 vs. VR 23	-5,805	-12,11 to 0,5016	No	ns	0,0785
VR 22 vs. MDP 22	-0,3500	-6,657 to 5,957	No	ns	>0,9999
VR 22 vs. MDP 23	-6,735	-13,04 to -0,4284	Yes	*	0,0340
VR 22 vs. HK 22	-28,38	-34,69 to -22,07	Yes	****	<0,0001
VR 22 vs. HK 23	-18,58	-24,89 to -12,27	Yes	****	<0,0001
VR 22 vs. LN 22	-11,29	-17,60 to -4,983	Yes	***	0,0008
VR 22 vs. LN 23	-20,01	-26,32 to -13,70	Yes	****	<0,0001
VR 22 vs. M 22	-12,35	-18,66 to -6,043	Yes	***	0,0004
VR 22 vs. M23	-49,28	-55,58 to -42,97	Yes	****	<0,0001
VR 23 vs. MDP 22	5,455	-0,8516 to 11,76	No	ns	0,1074
VR 23 vs. MDP 23	-0,9300	-7,237 to 5,377	No	ns	0,9997
VR 23 vs. HK 22	-22,58	-28,88 to -16,27	Yes	****	<0,0001
VR 23 vs. HK 23	-12,78	-19,08 to -6,468	Yes	***	0,0003
VR 23 vs. LN 22	-5,485	-11,79 to 0,8216	No	ns	0,1045
VR 23 vs. LN 23	-14,21	-20,51 to -7,898	Yes	***	0,0001
VR 23 vs. M 22	-6,545	-12,85 to -0,2384	Yes	*	0,0403
VR 23 vs. M23	-43,47	-49,78 to -37,16	Yes	****	<0,0001
MDP 22 vs. MDP 23	-6,385	-12,69 to -0,07838	Yes	*	0,0466
MDP 22 vs. HK 22	-28,03	-34,34 to -21,72	Yes	****	<0,0001
MDP 22 vs. HK 23	-18,23	-24,54 to -11,92	Yes	****	<0,0001
MDP 22 vs. LN 22	-10,94	-17,25 to -4,633	Yes	**	0,0010
MDP 22 vs. LN 23	-19,66	-25,97 to -13,35	Yes	****	<0,0001
MDP 22 vs. M 22	-12,00	-18,31 to -5,693	Yes	***	0,0005
MDP 22 vs. M23	-48,93	-55,23 to -42,62	Yes	****	<0,0001
MDP 23 vs. HK 22	-21,65	-27,95 to -15,34	Yes	****	<0,0001
MDP 23 vs. HK 23	-11,85	-18,15 to -5,538	Yes	***	0,0005
MDP 23 vs. LN 22	-4,555	-10,86 to 1,752	No	ns	0,2331
MDP 23 vs. LN 23	-13,28	-19,58 to -6,968	Yes	***	0,0002
MDP 23 vs. M 22	-5,615	-11,92 to 0,6916	No	ns	0,0931
MDP 23 vs. M23	-42,54	-48,85 to -36,23	Yes	****	<0,0001
HK 22 vs. HK 23	9,800	3,493 to 16,11	Yes	**	0,0025
HK 22 vs. LN 22	17,09	10,78 to 23,40	Yes	****	<0,0001
HK 22 vs. LN 23	8,370	2,063 to 14,68	Yes	**	0,0081
HK 22 vs. M 22	16,03	9,723 to 22,34	Yes	****	<0,0001
HK 22 vs. M23	-20,90	-27,20 to -14,59	Yes	****	<0,0001
HK 23 vs. LN 22	7,290	0,9834 to 13,60	Yes	*	0,0207
HK 23 vs. LN 23	-1,430	-7,737 to 4,877	No	ns	0,9928
HK 23 vs. M 22	6,230	-0,07662 to 12,54	No	ns	0,0536
HK 23 vs. M23	-30,70	-37,00 to -24,39	Yes	****	<0,0001
LN 22 vs. LN 23	-8,720	-15,03 to -2,413	Yes	**	0,0060
LN 22 vs. M 22	-1,060	-7,367 to 5,247	No	ns	0,9992
LN 22 vs. M23	-37,99	-44,29 to -31,68	Yes	****	<0,0001
LN 23 vs. M 22	7,660	1,353 to 13,97	Yes	*	0,0150
LN 23 vs. M23	-29,27	-35,57 to -22,96	Yes	****	<0,0001
M 22 vs. M23	-36,93	-43,23 to -30,62	Yes	****	<0,0001

Table S4a: Statistical analysis carried out on L* parameter of chicken-based burgers (Tukey's multiple comparisons test) characteristics of chicken-based burgers, prepared with 100, 70, and 50% chicken meat, without (control) and with 4% PPP (HK, Hokkaido variety)

Tukey's multiple comparisons test	Mean Diff,	95,00% CI of diff,	Below threshold?	Summary	Adjusted P Value	
Control 100 vs. Control 70:30	6,553	6,514 to 6,593	Yes	****	<0,0001	A-B
Control 100 vs. Control 50:50	9,320	9,281 to 9,359	Yes	****	<0,0001	A-C
Control 100 vs. Hamb HK 100	7,510	7,471 to 7,549	Yes	****	<0,0001	A-D
Control 100 vs. Hamb HK 70:30	13,38	13,34 to 13,42	Yes	****	<0,0001	A-E
Control 100 vs. Hamb HK 50:50	11,22	11,18 to 11,26	Yes	****	<0,0001	A-F
Control 70:30 vs. Control 50:50	2,767	2,727 to 2,806	Yes	****	<0,0001	B-C
Control 70:30 vs. Hamb HK 100	0,9567	0,9173 to 0,9960	Yes	****	<0,0001	B-D
Control 70:30 vs. Hamb HK 70:30	6,827	6,787 to 6,866	Yes	****	<0,0001	B-E
Control 70:30 vs. Hamb HK 50:50	4,670	4,631 to 4,709	Yes	****	<0,0001	B-F
Control 50:50 vs. Hamb HK 100	-1,810	-1,849 to -1,771	Yes	****	<0,0001	C-D
Control 50:50 vs. Hamb HK 70:30	4,060	4,021 to 4,099	Yes	****	<0,0001	C-E
Control 50:50 vs. Hamb HK 50:50	1,903	1,864 to 1,943	Yes	****	<0,0001	C-F
Hamb HK 100 vs. Hamb HK 70:30	5,870	5,831 to 5,909	Yes	****	<0,0001	D-E
Hamb HK 100 vs. Hamb HK 50:50	3,713	3,674 to 3,753	Yes	****	<0,0001	D-F
Hamb HK 70:30 vs. Hamb HK 50:50	-2,157	-2,196 to -2,117	Yes	****	<0,0001	E-F

Table S4b: Statistical analysis carried out on a* parameter of chicken-based burgers (Tukey's multiple comparisons test) characteristics of chicken-based burgers, prepared with 100, 70, and 50% chicken meat, without (control) and with 4% PPP (HK, Hokkaido variety)

Tukey's multiple comparisons test	Mean Diff.	95,00% CI of diff.	Below threshold?	Summary	Adjusted P Value	
Control 100 vs. Control 70:30	-6,677	-6,730 to -6,623	Yes	****	<0,0001	A-B
Control 100 vs. Control 50:50	-8,313	-8,367 to -8,260	Yes	****	<0,0001	A-C
Control 100 vs. Hamb HK 100	-2,360	-2,413 to -2,307	Yes	****	<0,0001	A-D
Control 100 vs. Hamb HK 70:30	-10,67	-10,72 to -10,62	Yes	****	<0,0001	A-E
Control 100 vs. Hamb HK 50:50	-9,810	-9,863 to -9,757	Yes	****	<0,0001	A-F
Control 70:30 vs. Control 50:50	-1,637	-1,690 to -1,583	Yes	****	<0,0001	B-C
Control 70:30 vs. Hamb HK 100	4,317	4,263 to 4,370	Yes	****	<0,0001	B-D
Control 70:30 vs. Hamb HK 70:30	-3,993	-4,047 to -3,940	Yes	****	<0,0001	B-E
Control 70:30 vs. Hamb HK 50:50	-3,133	-3,187 to -3,080	Yes	****	<0,0001	B-F
Control 50:50 vs. Hamb HK 100	5,953	5,900 to 6,007	Yes	****	<0,0001	C-D
Control 50:50 vs. Hamb HK 70:30	-2,357	-2,410 to -2,303	Yes	****	<0,0001	C-E
Control 50:50 vs. Hamb HK 50:50	-1,497	-1,550 to -1,443	Yes	****	<0,0001	C-F
Hamb HK 100 vs. Hamb HK 70:30	-8,310	-8,363 to -8,257	Yes	****	<0,0001	D-E
Hamb HK 100 vs. Hamb HK 50:50	-7,450	-7,503 to -7,397	Yes	****	<0,0001	D-F
Hamb HK 70:30 vs. Hamb HK 50:50	0,8600	0,8067 to 0,9133	Yes	****	<0,0001	E-F

Table S4c: Statistical analysis carried out on b* parameter of chicken-based burgers (Tukey's multiple comparisons test) characteristics of chicken-based burgers, prepared with 100, 70, and 50% chicken meat, without (control) and with 4% PPP (HK, Hokkaido variety)

Tukey's multiple comparisons test	Mean Diff,	95,00% CI of diff,	Below threshold?	Summary	Adjusted P Value	
Control 100 vs. Control 70:30	2,463	2,389 to 2,538	Yes	****	<0,0001	A-B
Control 100 vs. Control 50:50	5,797	5,722 to 5,871	Yes	****	<0,0001	A-C
Control 100 vs. Hamb HK 100	-8,107	-8,181 to -8,032	Yes	****	<0,0001	A-D
Control 100 vs. Hamb HK 70:30	-15,41	-15,48 to -15,34	Yes	****	<0,0001	A-E
Control 100 vs. Hamb HK 50:50	-17,10	-17,17 to -17,03	Yes	****	<0,0001	A-F
Control 70:30 vs. Control 50:50	3,333	3,259 to 3,408	Yes	****	<0,0001	B-C
Control 70:30 vs. Hamb HK 100	-10,57	-10,64 to -10,50	Yes	****	<0,0001	B-D
Control 70:30 vs. Hamb HK 70:30	-17,87	-17,95 to -17,80	Yes	****	<0,0001	B-E
Control 70:30 vs. Hamb HK 50:50	-19,56	-19,64 to -19,49	Yes	****	<0,0001	B-F
Control 50:50 vs. Hamb HK 100	-13,90	-13,98 to -13,83	Yes	****	<0,0001	C-D
Control 50:50 vs. Hamb HK 70:30	-21,21	-21,28 to -21,13	Yes	****	<0,0001	C-E
Control 50:50 vs. Hamb HK 50:50	-22,90	-22,97 to -22,82	Yes	****	<0,0001	C-F
Hamb HK 100 vs. Hamb HK 70:30	-7,303	-7,378 to -7,229	Yes	****	<0,0001	D-E
Hamb HK 100 vs. Hamb HK 50:50	-8,993	-9,068 to -8,919	Yes	****	<0,0001	D-F
Hamb HK 70:30 vs. Hamb HK 50:50	-1,690	-1,765 to -1,615	Yes	****	<0,0001	E-F
