# Supplementary material for: Development of Value-Added Chicken Burgers by Adding Pumpkin Peel Powder as a Sustainable Ingredient
Source: Antioxidants (Basel). 2025 May 28;14(6):648. doi: 10.3390/antiox14060648 (PMC12190050; doi:10.3390/antiox14060648)
Supplement: Supplementary file 1 [file antioxidants-14-00648-s001.zip › supplementary figures.pdf]

Development of value-added chicken burgers by adding pumpkin peel as a sustainable ingredient

Nicola Pinna [1,†], Federica Ianni [1,†], Michela Codini [1], Beniamino Cenci-Goga [2,3], Marco Misuraca [2], Egidia Costanzi [2], Lina Cossignani [1,*] and Francesca Blasi [1]

[1] Department of Pharmaceutical Sciences, University of Perugia, 06126 Perugia, Italy; nicola.pinna@dottorandi.unipg.it (N.P.); federica.ianni@unipg.it (F.I.); michela.codini@unipg.it (M.C.); francesca.blasi@unipg.it (F.B.)

[2] Department of Veterinary Medicine, University of Perugia, 06126 Perugia, Italy; beniamino.cencigoga@unipg.it (B.T.C.-G.); marco.misuraca.tdp@gmail.com (M.M.); egidia.costanzi@unipg.it (E.C.)

[3] Faculty of Veterinary Science, Department of Paraclinical Sciences, University of Pretoria, Onderstepoort 0110, South Africa

* Correspondence: lina.cossignani@unipg.it (L.C.); Tel.: +39-075-585-7959

† These authors contributed equally to this work.

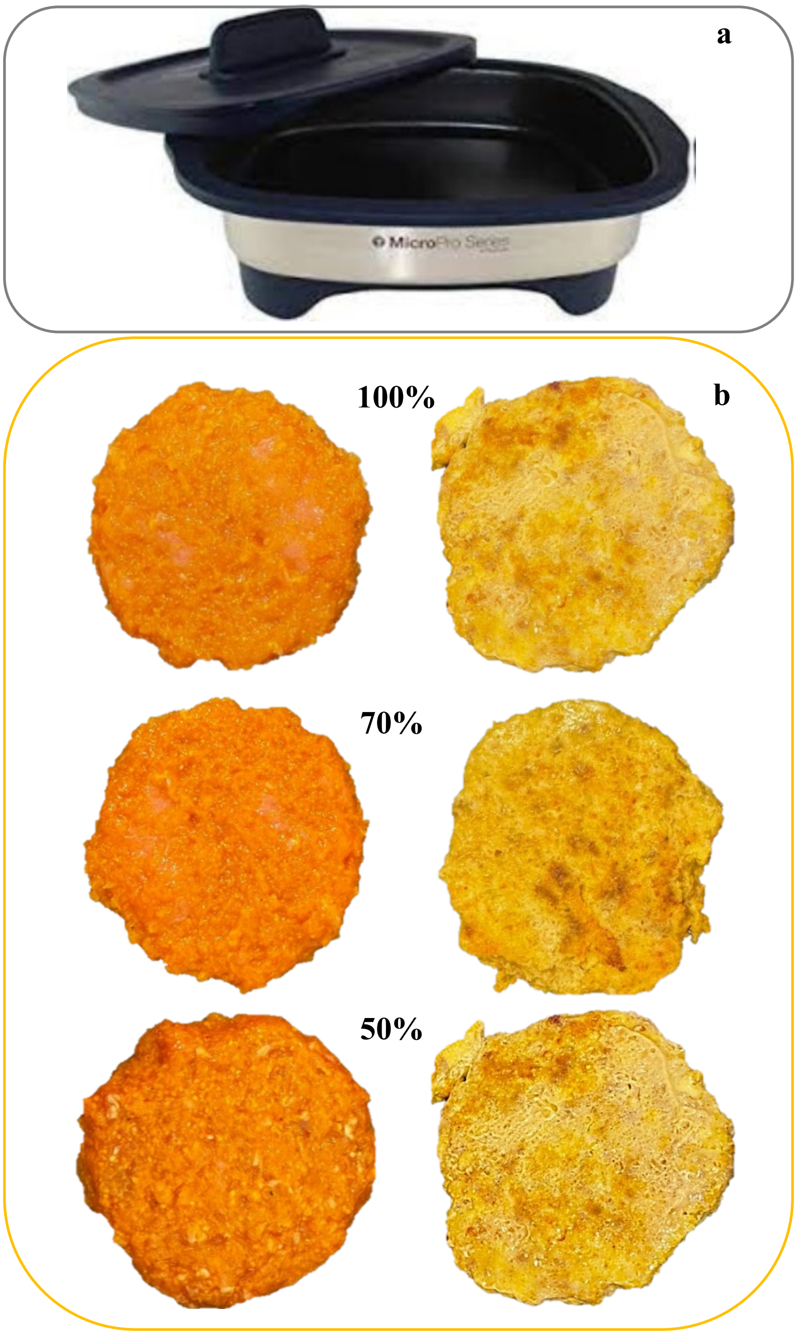

Figure S1. Cooking equipment (a), chicken-based burgers before and after cooking (b) with different percentages of chicken meat: 100, 70, and 50%.

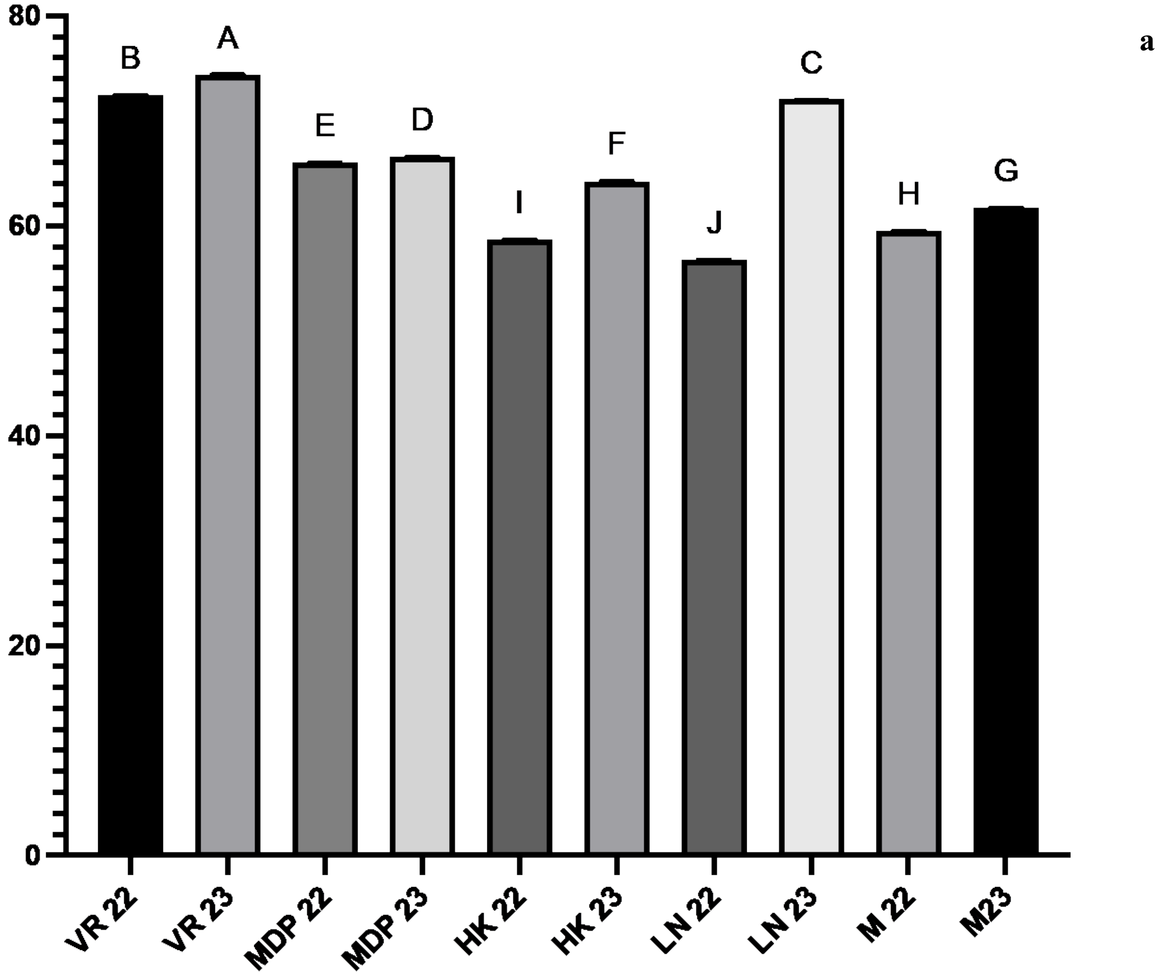

Figure S2a: Values of L* parameter of carotenoid extracts from PPP obtained from pumpkin harvested in 2022 and 2023 (mean values ± SD, n = 3). Different letters indicate significant differences with p-value < 0.01. (LN, Lunga di Napoli; MDP, Moscata di Provenza; VR, Violina rugosa; HK, Hokkaido; M, Mantovana) and harvesting years (2022-2023)

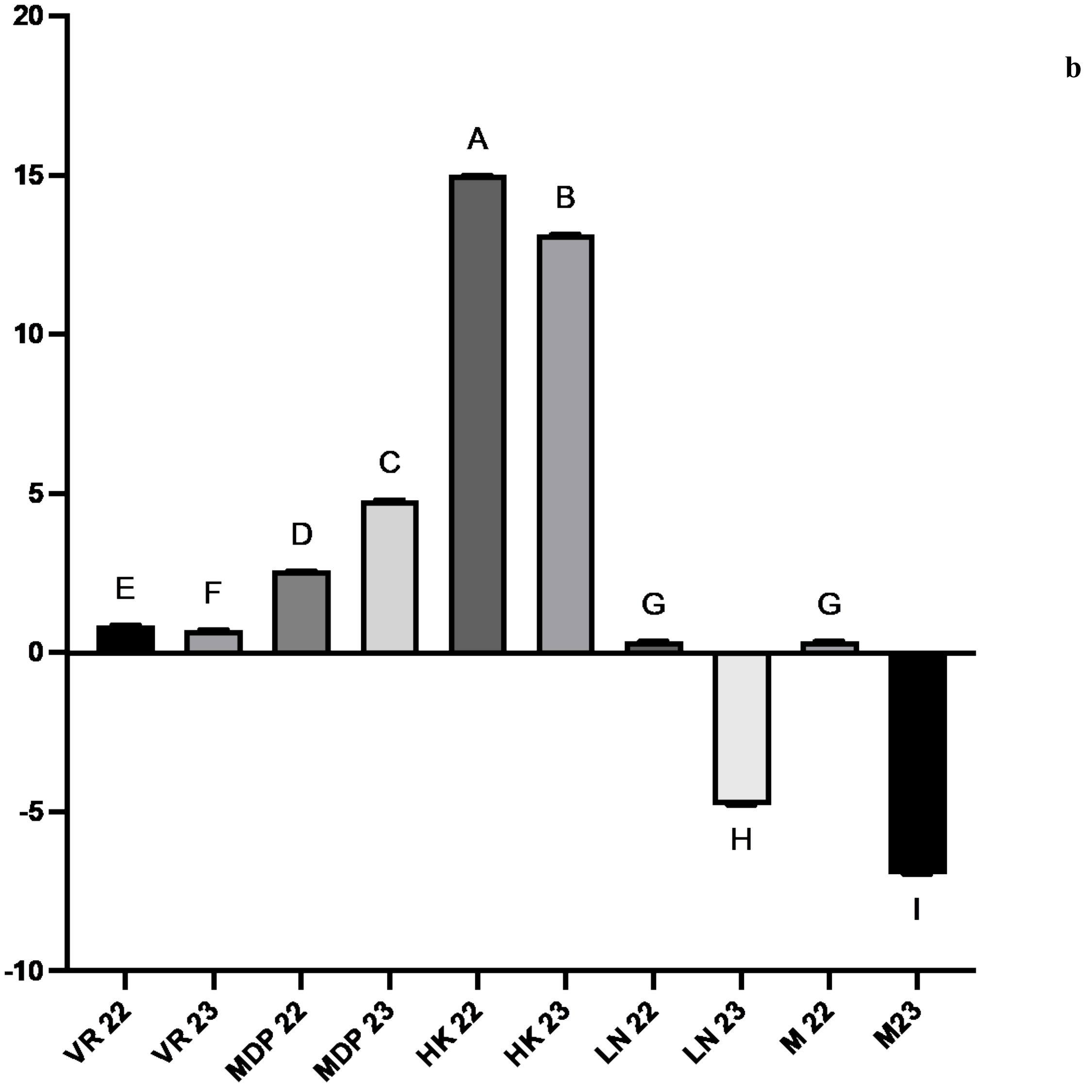

Figure S2b: Values of a* parameter of values of carotenoid extracts from PPP obtained from pumpkin harvested in 2022 and 2023 (mean values ± SD, n = 3). Different letters indicate significant differences with p-value < 0.01. (LN, Lunga di Napoli; MDP, Moscata di Provenza; VR, Violina rugosa; HK, Hokkaido; M, Mantovana) and harvesting years (2022-2023)

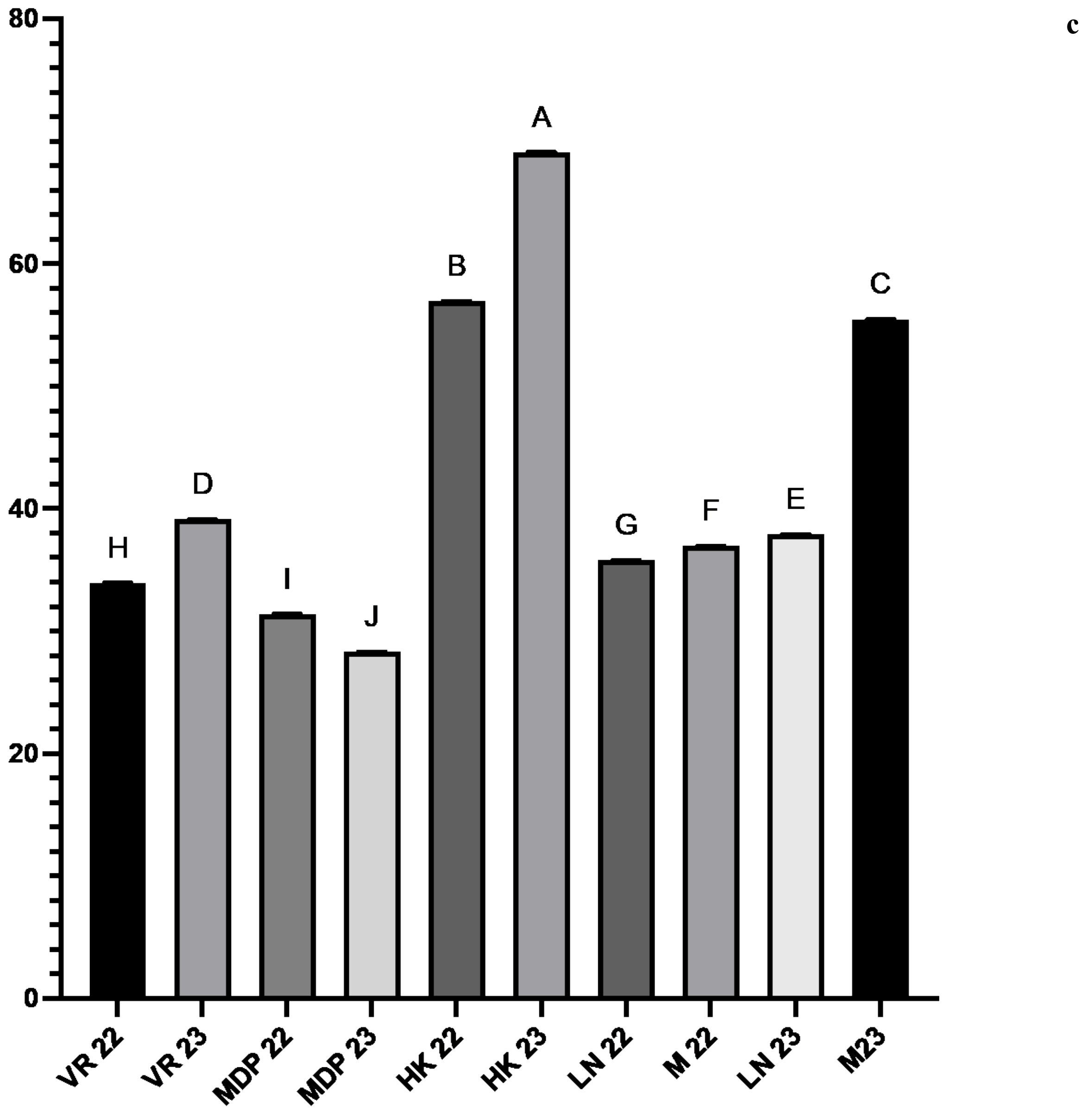

Figure S2c: Values of b* parameter of values of carotenoid extracts from PPP obtained from pumpkin harvested in 2022 and 2023 (mean values ± SD, n = 3). Different letters indicate significant differences with p-value < 0.01. (LN, Lunga di Napoli; MDP, Moscata di Provenza; VR, Violina rugosa; HK, Hokkaido; M, Mantovana) and harvesting years (2022-2023)

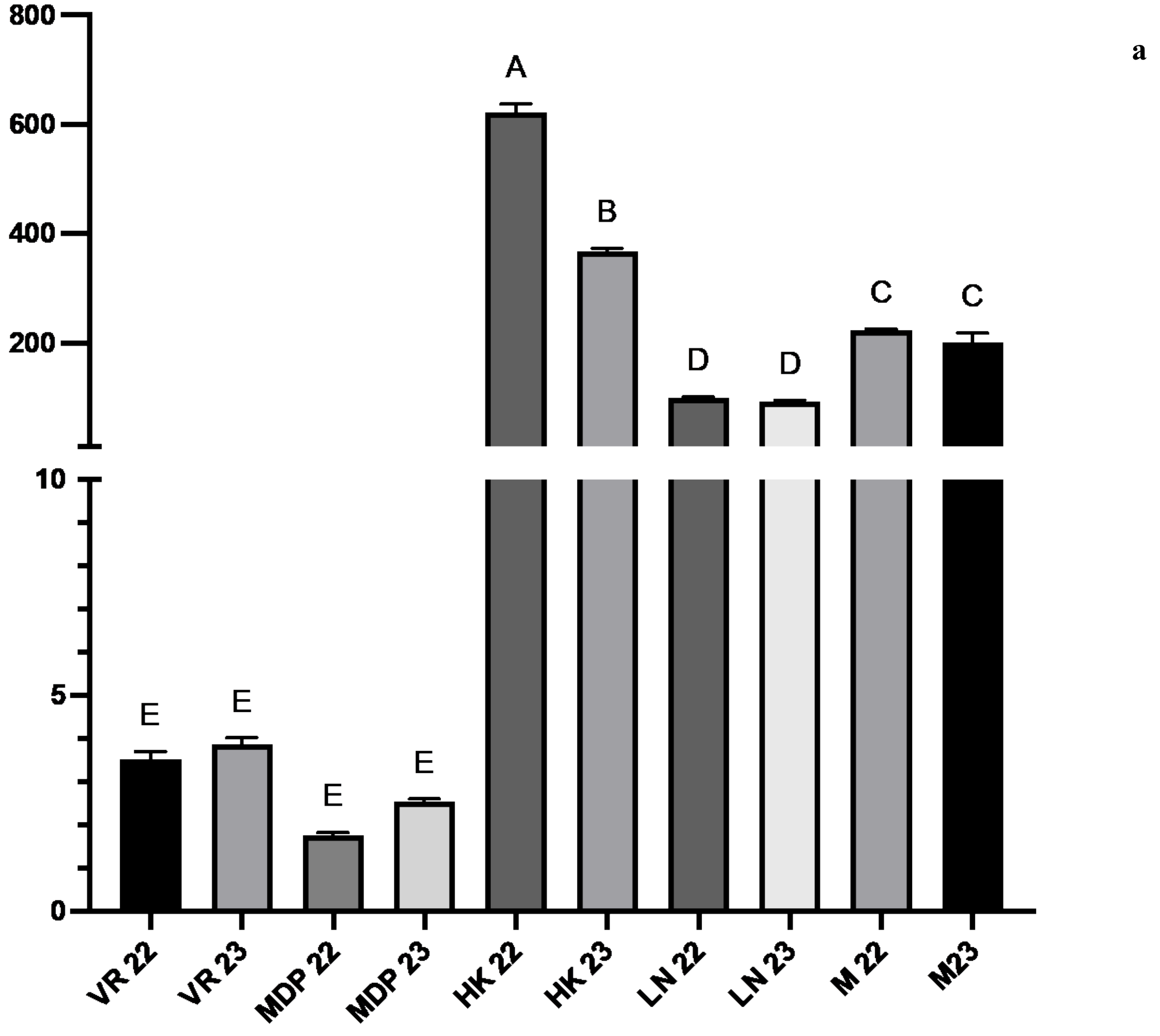

Figure S3a: Values (µg LE/g) of free xanthophylls of extracts from PPP obtained from pumpkin harvested in 2022 and 2023 (mean values ± SD, n = 3). Different letters indicate significant differences with p-value < 0.01. (LN, Lunga di Napoli; MDP, Moscata di Provenza; VR, Violina rugosa; HK, Hokkaido; M, Mantovana) and harvesting years (2022-2023). LE, Lutein Equivalents.

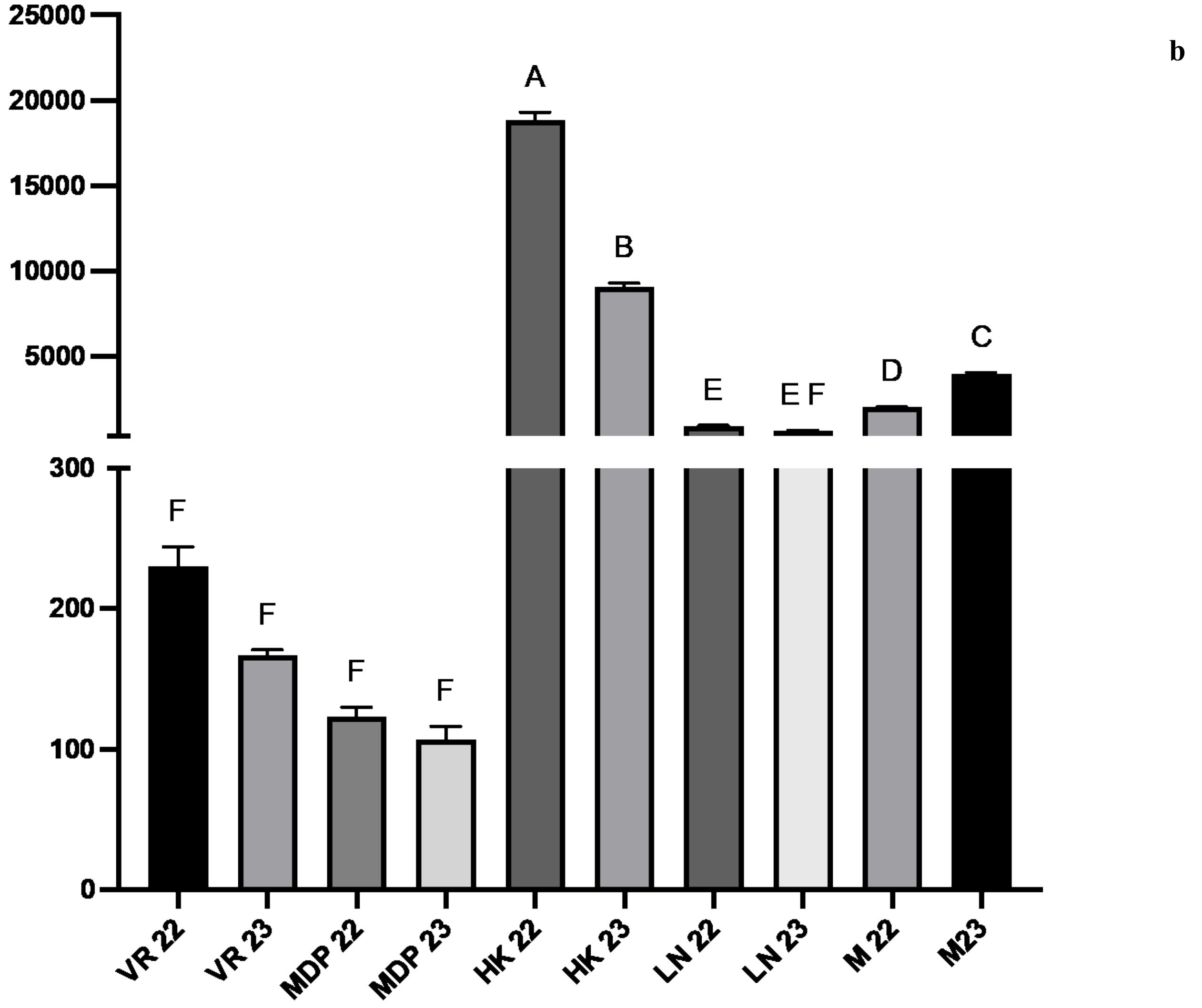

Figure S3b: Values (µg ZDE/g) of monoesterified xanthophylls of extracts from PPP obtained from pumpkin harvested in 2022 and 2023 (mean values ± SD, n = 3). Different letters indicate significant differences with p-value < 0.01. (LN, Lunga di Napoli; MDP, Moscata di Provenza; VR, Violina rugosa; HK, Hokkaido; M, Mantovana) and harvesting years (2022-2023). ZDE, Zeaxanthin Dipalmitate Equivalents

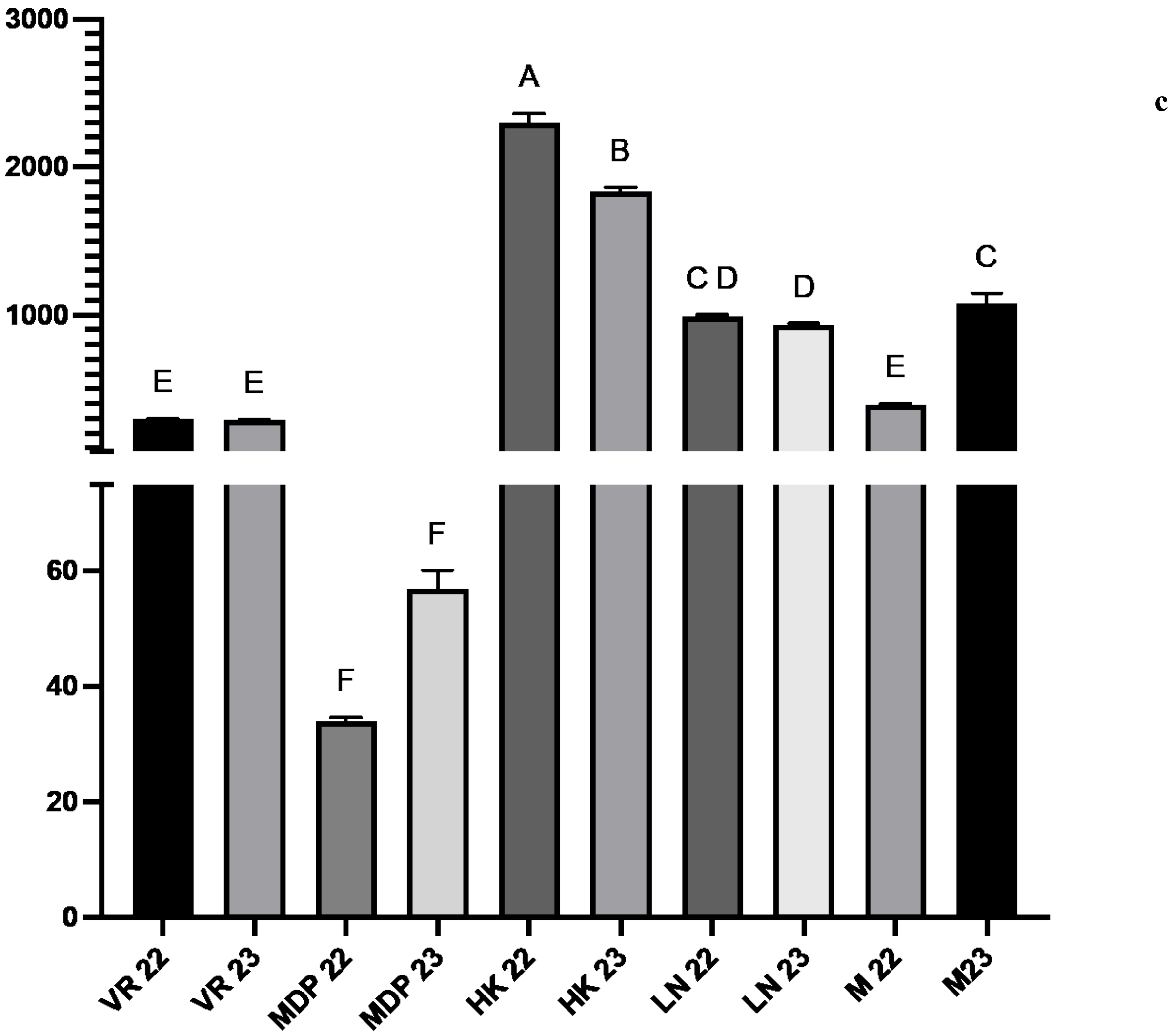

Figure S3c: Values (µg ZDE/g) of disterified xanthophylls of extracts from PPP obtained from pumpkin harvested in 2022 and 2023 (mean values ± SD, n = 3). Different letters indicate significant differences with p-value < 0.01. (LN, Lunga di Napoli; MDP, Moscata di Provenza; VR, Violina rugosa; HK, Hokkaido; M, Mantovana) and harvesting years (2022-2023). ZDE, Zeaxanthin Dipalmitate Equivalents.

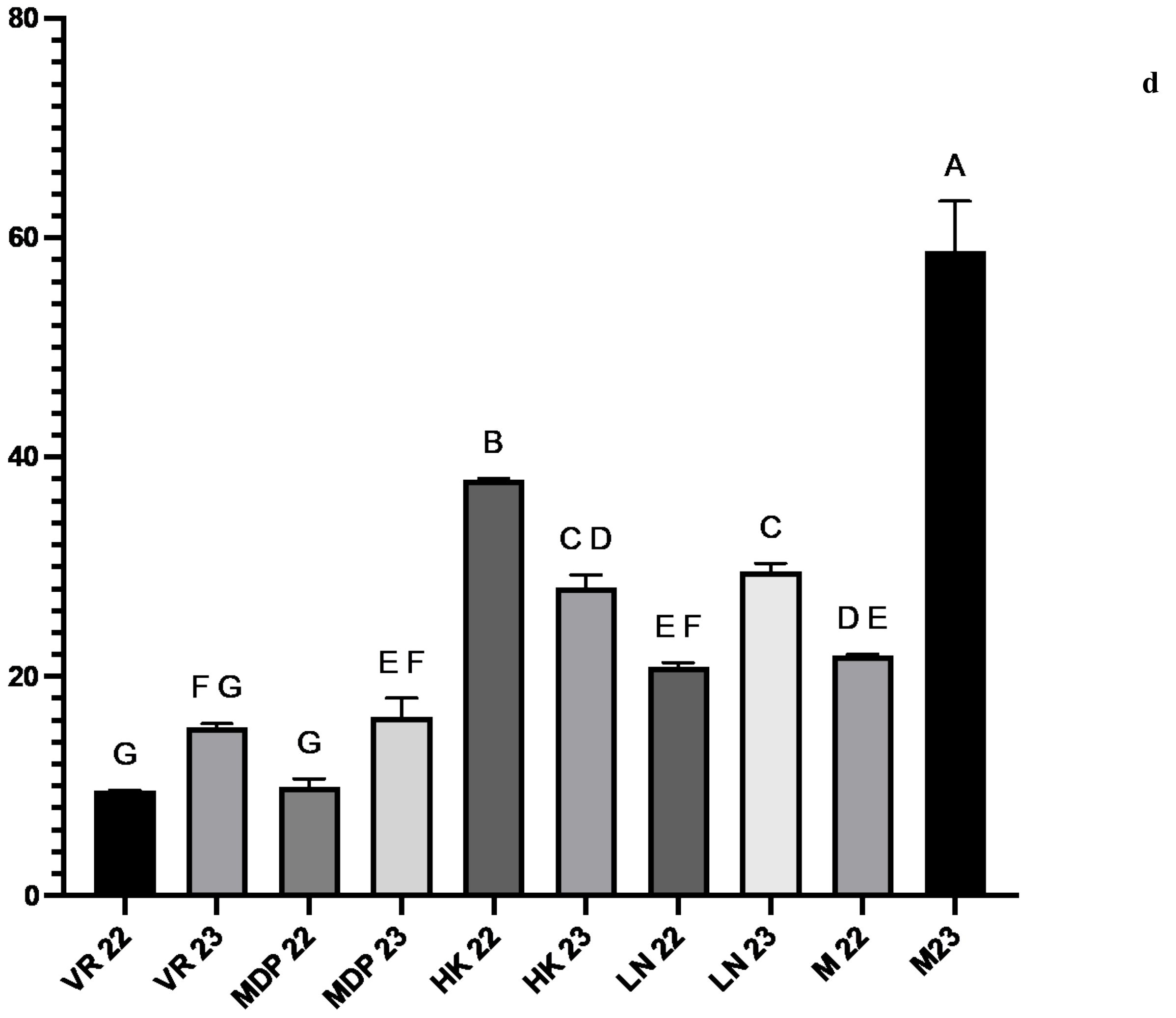

Figure S3d: Values (µg/g) of β-carotene of extracts from PPP obtained from pumpkin harvested in 2022 and 2023 (mean values ± SD, n = 3). Different letters indicate significant differences with p-value < 0.01. (LN, Lunga di Napoli; MDP, Moscata di Provenza; VR, Violina rugosa; HK, Hokkaido; M, Mantovana) and harvesting years (2022-2023).

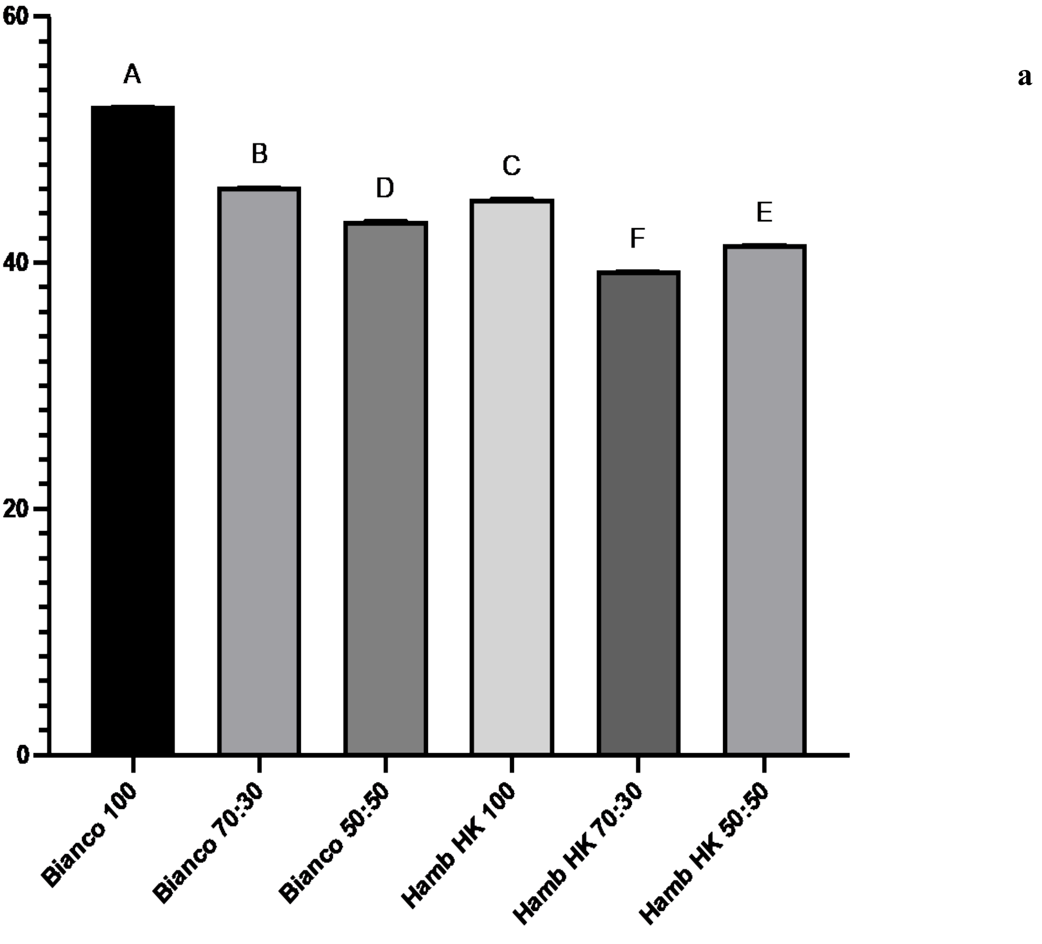

Figure S4a: Values (mean values ± SD, n = 3) of L* parameter of chicken-based burgers made with 100, 70, and 50% chicken meat, without (control) and with 4% PPP (HK, Hokkaido variety). Different letters indicate significant differences with p-value < 0.01. HK, Hokkaido.

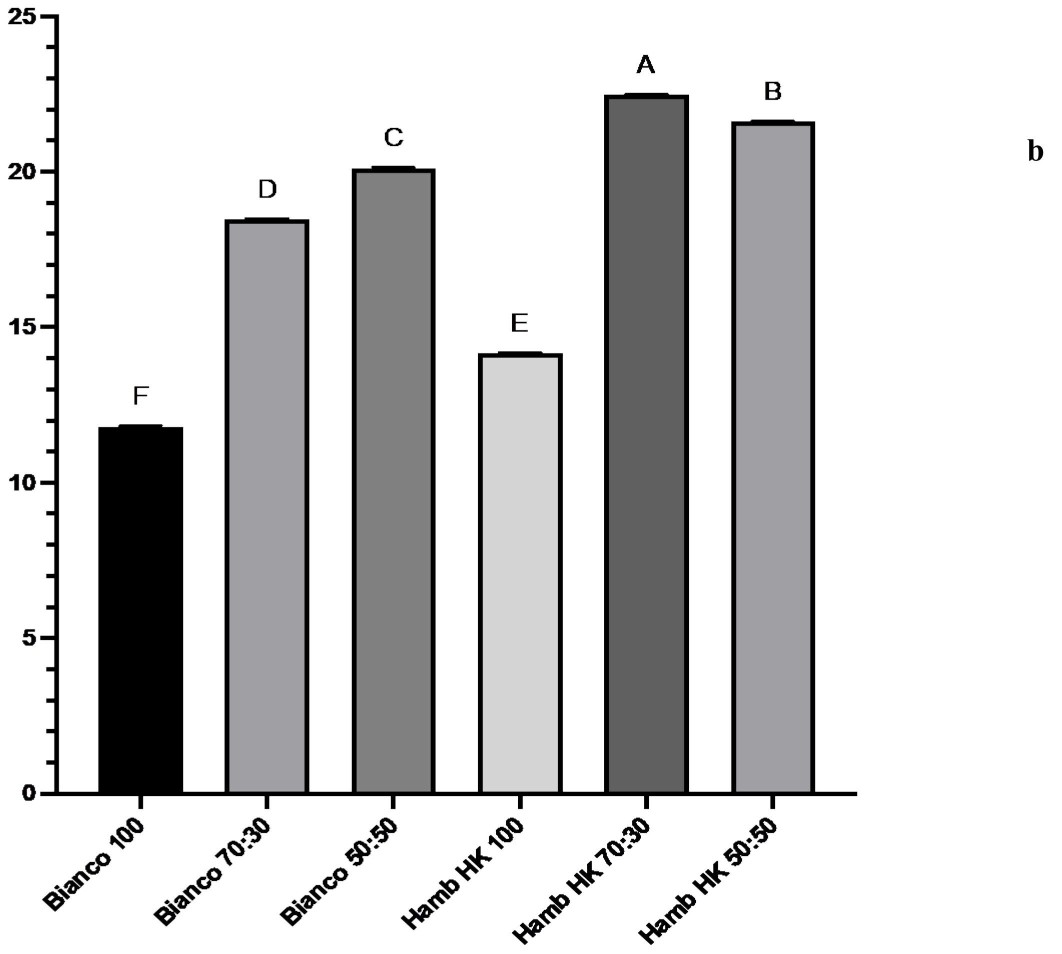

Figure S4b: Values (mean values ± SD, n = 3) of a* parameter of chicken-based burgers made with 100, 70, and 50% chicken meat, without (control) and with 4% PPP (HK, Hokkaido variety). Different letters indicate significant differences with p-value < 0.01. HK, Hokkaido.

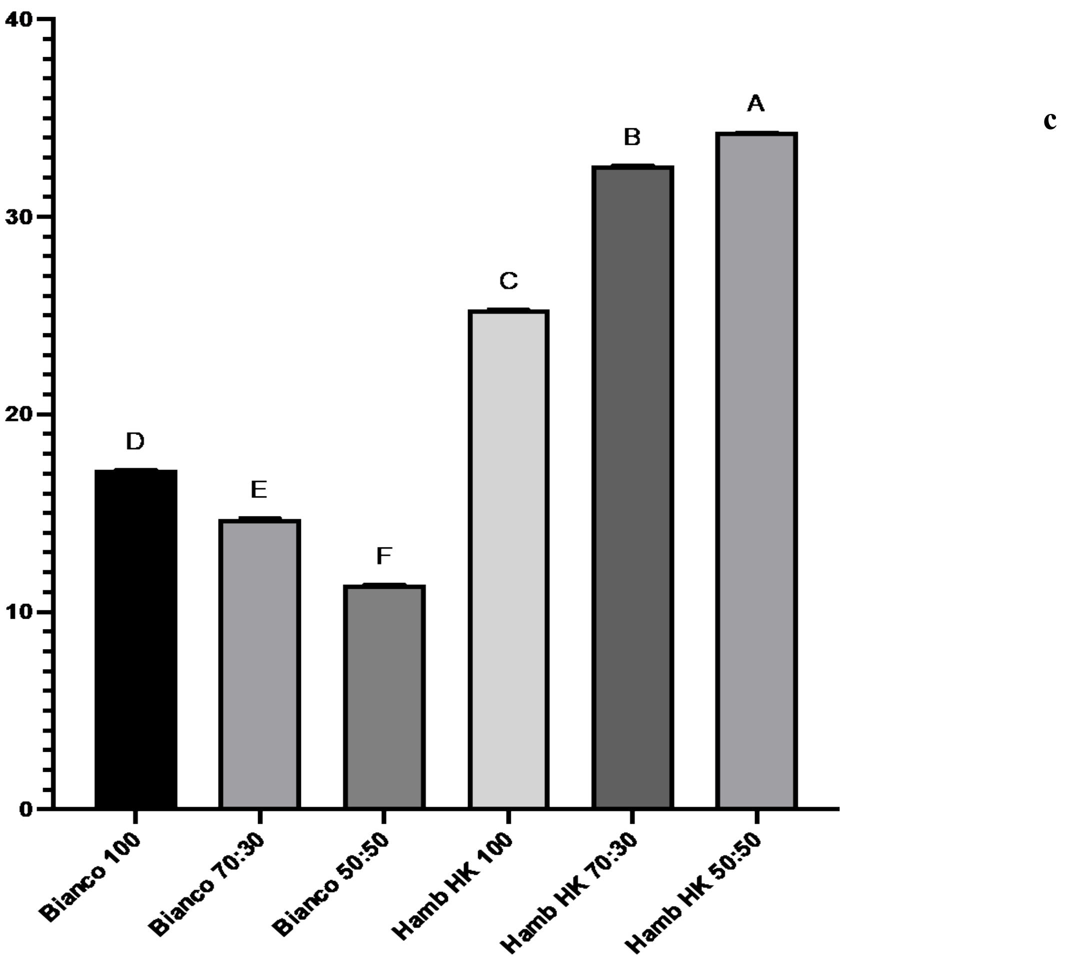

Figure S4c: Values (mean values ± SD, n = 3) of b* parameter of chicken-based burgers made with 100, 70, and 50% chicken meat, without (control) and with 4% PPP (HK, Hokkaido variety). Different letters indicate significant differences with p-value < 0.01. HK, Hokkaido.
